# COVID-19 Education for Health Professionals Caring for Spanish-Speaking Patients

**DOI:** 10.15766/mep_2374-8265.11240

**Published:** 2022-04-12

**Authors:** Anibelky Almanzar, Derek Martinez, Edgar Vega, Miguel Lopez, Linda Hodes-Villamar, John Paul Sánchez

**Affiliations:** 1 Fourth-Year Medical Student, Northeast Ohio Medical University College of Medicine; 2 First-Year Resident, Department of Emergency Medicine, University of Oklahoma College of Medicine; 3 Fourth-Year Medical Student, University of California, San Diego, School of Medicine; 4 Fourth-Year Pharmacy Student, University of New Mexico College of Pharmacy; 5 Clerkship Director and Assistant Professor, Department of Emergency Medicine, University of New Mexico School of Medicine; 6 Executive Associate Vice Chancellor, Diversity, Equity and Inclusion, Health Sciences Center, University of New Mexico School of Medicine

**Keywords:** Medical Spanish, COVID-19, Virtual Learning, Diversity, Inclusion, Health Equity

## Abstract

**Introduction:**

The COVID-19 pandemic has disproportionately affected Hispanics in the United States, who make up 18% of US inhabitants but 29% of COVID-19 cases as of June 2021. Recent studies have attributed higher COVID-19 infection, hospitalization, and death rates among Hispanics to social determinants of health. Given that the majority of US Hispanics are bilingual or Spanish-dominant, it is imperative for health care providers to be prepared to discuss COVID-19 prevention and treatment in Spanish.

**Methods:**

We developed an interactive workshop aimed at increasing health professionals' confidence in discussing COVID-19 prevention, risk factors, and treatments with Spanish-speaking patients. Learners were expected to have an intermediate level or higher proficiency in medical Spanish. The workshop consisted of a PowerPoint presentation and English/Spanish scripts to facilitate interactive learning. The workshop was evaluated using a postworkshop questionnaire to assess learners' perceived confidence in communicating with Spanish-speaking patients.

**Results:**

The workshop was implemented with 70 participants, who had diverse ethnoracial identities and professional roles, at five different medical schools. Fifty-three participants completed the postworkshop questionnaire. More than 50% reported near complete to complete confidence in meeting the three learning objectives.

**Discussion:**

With Hispanics being the largest non-White ethnoracial group in the US and being disproportionally affected by COVID-19, it is essential for health professionals to access training tools that allow them to practice medical Spanish. This module can uniquely aid in the preparation of health professionals caring for Spanish-speaking patients who present with COVID-19 symptoms.

## Educational Objectives

By the end of this activity, learners will be able to:

1. Discuss the diagnosis, symptoms, treatment, and vaccines associated with COVID-19 with a Spanish-speaking patient.
2. Compare the epidemiology of COVID-19 regarding race and ethnicity.
3. Discuss a plan to discharge home versus admission to the hospital with a Spanish-speaking patient.

## Introduction

In 2020, Hispanic communities made up 18% of the US population^1^; however, as of June 2021, Hispanics made up 29% of COVID-19 cases. In comparing ratios between White non-Hispanic and Hispanic persons, Hispanic persons are twice as likely to have COVID-19 infection, almost three times more likely to be hospitalized, and almost twice as likely to die from COVID-19 or associated complications.^2^

Recent studies hypothesize that higher COVID-19 infection and hospitalization among Hispanics may be due to social determinants of health.^3^ Social determinants of health are defined by the World Health Organization as “the nonmedical factors that influence health outcomes. They are the conditions in which people are born, grow, work, live, and age.”^4^ Minoritized groups are more likely to have gaps in health literacy, communication with medical providers, and access to medical care.^5^ Language barriers have disproportionately contributed to the Hispanic community's inability to access health-related information on how to stay safe during the COVID-19 pandemic.^6^ An analysis of language barriers in health care settings showed that people with limited English proficiency are less likely to have primary care visits and less likely to utilize preventative care.^7^ Also, according to Ortega and colleagues, “language has been identified as a major contributor to health disparities for patients who prefer languages besides English and may result in an increased risk of medical errors and poor health outcomes.”^8^ In 2015, the American Community Survey determined that out of the 40 million Spanish-speaking Hispanic US residents, 17.2 million speak only Spanish.^9^ Census 2017 data show that 72% of Hispanics speak a language other than English at home.^10^ At the beginning of the COVID-19 pandemic, information about the virus was available only in English, which may have further exacerbated the impact of the pandemic among Spanish-speaking individuals.^11^ Additionally, Jacobs and colleagues point out that “Latinos who speak Spanish have been shown to be less satisfied with their communication with health care providers, the care they receive, and more likely to report overall problems with care than are English speakers.”^12^

In *MedEdPORTAL,* there are a few medical Spanish modules for health professionals and medical trainees, but none specifically focus on the symptoms, epidemiology, treatment, and vaccines associated with COVID-19. This module was developed for health professionals (e.g., physicians-in-training) with a perceived intermediate or higher level of medical Spanish proficiency. This workshop provides a unique opportunity for clinicians and trainees to practice communicating about COVID-19 signs, symptoms, epidemiology, and treatment with Spanish-speaking patients. The workshop addresses specific questions Spanish-speaking patients may have about COVID-19, including information about the most common vaccines. The Spanish and English scripts give learners an opportunity to practice a patient-physician encounter and the ensuing discussion regarding mild and severe COVID-19 infection.

This workshop was developed and evaluated by health professional students from across the US identifying as Latina/o/x, Hispanic, or of Spanish origin, with oversight from Dr. John Paul Sánchez and Dr. Linda Hodes-Villamar. Dr. Sánchez is of Puerto Rican ancestry and is an emergency medicine physician with 20 years of experience in developing and implementing curricula for health care and public health learners. Dr. Hodes-Villamar is of Mexican ancestry and is an emergency medicine physician and student clerkship director at the University of New Mexico's emergency medicine residency program. Dr. Hodes-Villamar is recognized for her understanding of how language and other socioeconomic disparities affect patients in the emergency department. Anibelky Almanzar is a fourth-year medical student at the Northeast Ohio Medical University College of Medicine. She grew up in the Dominican Republic and is deeply passionate about working with underserved communities, especially with Spanish-speaking patients. Derek Martinez, DO, is an emergency medicine resident physician at the University of Oklahoma. He is a first-generation Mexican American whose family moved to the United States from Guanajuato, Mexico. Edgar Vega is a fourth-year medical student at the University of California, San Diego, School of Medicine. He is a first-generation Mexican American and has roots in Morelia, Michoacán, and El Llano, Queretaro. He is an aspiring emergency medicine physician. Miguel Lopez is a fourth-year pharmacy student at the University of New Mexico College of Pharmacy. He grew up in St. Petersburg, Florida. He lived in Morelia, Michoacán, for the first 3 years of elementary school and was immersed in the Spanish language and culture. All authors were active participants in developing, implementing, and evaluating the curriculum material.

## Methods

The six-step Kern model^13^ was applied as a framework for the design, implementation, and evaluation of the workshop. In terms of step 1: general needs assessment and step 2: targeted needs assessment, we performed a literature review and reviewed other medical Spanish submissions in *MedEdPORTAL.* We also gathered input from medical trainees who reported that discussing COVID-19 with Spanish-speaking patients was of increasing importance. For step 3: goals and objectives, we determined the goal of the workshop was to increase confidence when discussing COVID-19 with Spanish-speaking patients. The objectives included discussing the symptoms, diagnosis, treatment, and vaccines associated with COVID-19 with a Spanish-speaking patient; comparing the epidemiology of COVID-19 by race and ethnicity; and discussing discharge home or admission to the hospital with a Spanish-speaking patient. Regarding step 4: educational strategies, the workshop included an instructional PowerPoint presentation, two case-based educational videos in Spanish, two scripted cases in Spanish and English, and a postworkshop self-assessment. The module was developed for health professionals (e.g., physicians-in-training) with a perceived intermediate or higher level of medical Spanish proficiency. For step 5: implementation, the workshop was presented via Zoom at five sites: the Latino Medical Student Association National Conference; the Department of Emergency Medicine at the University of New Mexico; the University of California, Davis, School of Medicine; Texas A&M University College of Medicine; and Northeast Ohio Medical University. The workshop was implemented over 60- and 90-minute time frames. For step 6: evaluation and feedback, the postworkshop self-assessment was implemented via Google Forms. The form collected learners' self-perceived confidence in communicating with Spanish-speaking patients about COVID-19 after the workshop.

The ideal facilitator(s) of this workshop should have advanced-level Spanish proficiency, some clinical background regarding COVID-19, and an understanding of how social determinants of health impact the health of the Hispanic community. The facilitator guide (Appendix A) offers a step-by-step approach to implementing the workshop. Materials required include audiovisual equipment, a computer for the PowerPoint presentation, and printed copies of postworkshop evaluation forms. The suggested timeline for the 60- or 90-minute workshop is as follows:

• PowerPoint presentation (Appendix B): 15 minutes.
• Optional case videos (Appendices C–F): 5–10 minutes (90-minute session only).
• Practice scripts (Appendix G): 25 minutes.
• Feedback: 10 minutes.
• Postworkshop evaluation (Appendix H): 2–5 minutes.

To help learners have a basic understanding of coronavirus, we suggest two additional readings that can be given to them prior to the workshop.^14,15^

The interactive workshop began with a 20-slide PowerPoint presentation (Appendix B). The PowerPoint included information about COVID-19 symptoms, epidemiology, treatment, and vaccines, with all information in English and Spanish. The facilitator guide (Appendix A) provided annotations for each slide in English and Spanish to ensure consistent delivery. Prior to practicing the scripts, the facilitator could choose to show the optional Spanish and English videos for Case 1 (Appendices C and D) and Case 2 (Appendices E and F) to provide auditory and visual learning opportunities for the scripts. All videos were author owned. For the 60-minute version of the session, learners were provided with two patient encounter scripts (Appendix G) directly following the didactic portion. The cases were in Spanish and English to give learners an opportunity to practice a patient encounter. Case 1 was a script for an encounter in which the patient could be discharged. Case 2 was a script for an encounter in which the patient was admitted. Learners used these scripts to familiarize themselves with important medical Spanish vocabulary.

Following the script practice, learners had an opportunity to provide verbal feedback on the session and then were given a postworkshop evaluation (Appendix H) to assess their self-perceived confidence with the material provided. The evaluation also allowed participants to highlight strengths of the workshop and provided feedback for improvement. We used IBM SPSS Statistics version 28.0 to run statistical analyses of the quantitative data.

## Results

A total of 70 participants attended the five workshops. Fifty-three medical students, residents, and/or faculty completed a postworkshop questionnaire. The questionnaire asked participants to use a 5-point Likert scale (0 = *no confidence,* 4 = *complete confidence*) to indicate how much confidence they had in their ability to meet each learning objective. Using SPSS, we calculated the mean score of participants' responses on the postworkshop questionnaire. For Educational Objective 1 (discuss the diagnosis, symptoms, treatment, and vaccines associated with COVID-19 with a Spanish-speaking patient), the mean score was 2.8; for Educational Objective 2 (compare the epidemiology of COVID-19 in regard to race and ethnicity), the mean score was 2.7; and for Educational Objective 3 (discuss a plan to discharge home versus admission to the hospital with a Spanish-speaking patient), the mean score was 2.7.

In response to the question “What did you like about the workshop?”, participants responded:

• “The script was a very interactive way to get everyone involved.”
• “I enjoyed the chance to learn about an important current topic in medicine and how we can comfortably expand it to the Spanish-speaking community.”
• “It was fun! I enjoyed getting to speak in Spanish with my peers about COVID and vaccines. Very relevant!”
• “It was appropriate for intermediate, advanced Spanish learners. Love that it was focused to COVID.”
• “Learned new words in Spanish.”

In addition, attendees were also asked to provide suggestions on how to improve the workshop. Below are a few of their comments:

• “Include vocabulary from different regions for common terminology.”
• “Include a common words and phrases guide.”
• “Another script about vaccine and vaccine hesitancy.”

Several participants enjoyed the hybrid model (Spanish and English) of the presentation and expressed interest in wanting more workshops with a similar format. Several noted the usefulness of a vocabulary sheet prior to the workshop.

## Discussion

In order to address the high levels of COVID-19 in the Hispanic community, this workshop was developed to teach intermediate- to advanced-level Spanish-speaking health professionals proper terminology and phrasing when interacting with Spanish-speaking patients presenting with COVID-19 symptoms. The workshop is unique in that the presentation describes the heavy health burden that the Hispanic and Spanish-speaking communities face in relation to COVID-19. By the end of the workshop, participants expressed moderate self-perceived confidence in discussing the symptoms, diagnosis, treatment, and vaccines associated with COVID-19 with Spanish-speaking patients; in comparing the epidemiology of COVID-19 by race and ethnicity; and in discussing discharge home or admission to the hospital plans with Spanish-speaking patients.

Participants' feedback demonstrated that the workshop content and format were effective in describing the disproportionate burden the COVID 19 pandemic has had on the Hispanic community. They noted that they underestimated how beneficial it would be to use cases to practice Spanish and build vocabulary. Additionally, they mentioned that it was useful to work as a team during the role-play exercises utilizing the two scripts. For example, if a participant did not know how to state a particular phrase or word in Spanish, it was beneficial to have peers or colleagues to provide suggestions.

Opportunities for improvement were obtained from the postworkshop questionnaire. In their feedback, participants highlighted the linguistic diversity of the Spanish language and the importance of including common terminology for the same word that may be used in various regions of Latin America. An example highlighted was the Spanish word for *itchy,* which can include *picor, comezón,* and *salpullido*. There was also a discussion regarding the Spanish script activity and how it could be utilized as a tool to elicit more conversation among the participants. They suggested we provide instructions to elicit a specific set of information such as the history of present illness and then have participants share the questions they created so that others can benefit from seeing various forms of obtaining the same information from the patient.

The workshop's content has been revised, and we have integrated feedback from each prior session. This includes a more refined and effective presentation that highlights pertinent information and is inclusive of the varying levels of patient health literacy. Other educators may want to build on this workshop by developing modules to address vaccine hesitancy.

There were several notable limitations with the implementation and evaluation of this workshop. We only implemented the workshop virtually and therefore have no evaluation data related to implementing it in person. Anecdotally, we believe an in-person session would provide a more engaging experience because it could allow for a greater level of participation and appreciation of verbal and body language. In addition, we only reported on postworkshop data, which did not allow participants to share increased levels of confidence. An alternate approach to showcasing increased confidence would have been through a pre- and postworkshop questionnaire. Lastly, we did not formally analyze the qualitative comments because of the sparse amount of data.

This workshop can be easily integrated into any medical Spanish curriculum at the medical school or residency training level. Even if a formal medical Spanish curriculum is not implemented, the comprehensive facilitator guide allows a native speaker or anyone with an advanced level of medical Spanish to lead the workshop. Additionally, the workshop can be utilized as a resource for individuals looking to expand on COVID-19 curricular content that specifically focuses on the Spanish-speaking Hispanic community.

## Appendices


Facilitator Guide.docxCOVID-19 Presentation.pptxSpanish Clinical Encounter for Case 1.mp4English Clinical Encounter for Case 1.mp4Spanish Clinical Encounter for Case 2.mp4English Clinical Encounter for Case 2.mp4English and Spanish Scripts for Cases 1 & 2.docxPostworkshop Evaluation.docx
*All appendices are peer reviewed as integral parts of the Original Publication.*
