# Supplementary material for: COVID-19 Education for Health Professionals Caring for Spanish-Speaking Patients
Source: MedEdPORTAL. 2022 Apr 12;18:11240. doi: 10.15766/mep_2374-8265.11240 (PMC9001760; doi:10.15766/mep_2374-8265.11240)
Supplement: Supplementary file 1 — Facilitator Guide.docxCOVID-19 Presentation.pptxSpanish Clinical Encounter for Case 1.mp4English Clinical Encounter for Case 1.mp4Spanish Clinical Encounter for Case 2.mp4English Clinical Encounter for Case 2.mp4English and Spanish Scripts for Cases 1 & 2.docxPostworkshop Evaluation.docx [file mep_2374-8265.11240-s001.zip › G. English and Spanish Scripts for Cases 1 & 2.docx]

**English Script: Case 1**

**Doctor**: (knocks on the door)

**Patient**: Go ahead

**Doctor**: Hello Carmen, I am doctor _________, I’ll be seeing you today. Tell me, what is the reason for your visit?

**Patient**: Doctor I have had a fever, and a cough and I’ve been very tired these past few days.

**Doctor**: Oh, I am very sorry that you are going through this. I hope I can help you. Tell me, when did your symptoms start?

**Patient**: Three days ago I had a dry cough and I took allergy medicine because I thought I had allergies. But then I started to feel very tired and feverish.

**Doctor**: When was the first time you noticed the fever?

**Patient**: Two days ago when my husband took my temperature and it was 102.5 degrees.

**Doctor**: And what did you do when you realized you had a fever?

**Patient**: I took motrin and laid down to sleep.

**Doctor**: Did you feel any improvement with motrin?

**Patient**: Yes, my fever went down for a few hours, but the next day I felt a fever again. Along with the cough and fatigue that do not go away.

**Doctor**: What do you think is wrong?

**Patient**: I really don't know. I am confused with the symptoms of allergies, the common cold, the flu, and the COVID-19 virus. Doctor, could you explain the difference?

**Doctor**: That’s a very good question Carmen. Allergies do not produce fever, are not contagious, and cause itchy eyes or itchy skin. The common cold may cause sore throat, runny nose, and cough. The flu and COVID-19 present similarly and it can be very difficult to differentiate them based just on symptoms.

Do you think you have been exposed to people who have COVID-19?

**Patient**: I really don't know. I always try to wear a mask that covers my mouth and nose. I have not traveled or been meeting with a lot of people. But my husband and I work and we are exposed.

Doctor, do you think I could have COVID?

**Doctor**: It is possible given your symptoms. But first I need to ask you more questions and then we need to do the screening tests to give you a definitive answer. Is that okay?

**Patient:** Yes, that's fine with me.

**Doctor**: Other than you and your husband, does anyone else live in your house?

**Patient**: No. It's just me and him.

**Doctor**: And does your husband also have the same symptoms as you?

**Patient**: No. He just complains that he cannot smell or taste food for a few days.

**Doctor**: Loss of smell and taste can be caused by COVID. It may be that you or your husband have been exposed to the virus without knowing it. Like your husband, have you experienced loss of smell and / or taste?

**Patient**: Yes

**Doctor**: When did you notice it?

**Patient**: I've been like this for a few days but I didn't pay much attention to it. I think three days ago.

**Doctor:** What do you do for work?

**Patient**: I work in childcare.

**Doctor:** Now I want to ask you other more specific questions to get a better idea of what is going on.

**Patient:** Ok

**Doctor:** Have you had a headache?

**Patient:** No

**Doctor:** Have you had a stuffy nose?

**Patient**: No

**Doctor**: Have you had a sore throat?

**Patient**: Yes. I think the sore throat started at the same time.

**Doctor**: Right now, do you feel short of breath?

**Patient**: No.

**Doctor**: And when you walk, do you feel short of breath?

**Patient**: No.

**Doctor:** Have you had chest pain?

**Patient**: No

**Doctor**: Have you had abdominal pain, vomiting, or diarrhea?

**Patient**: No

**Doctor:** Now I would like to know more about your medical history. What medical conditions do you have?

**Patient**: I have type 2 diabetes, high blood pressure and high cholesterol.

**Doctor**: What medicines do you take?

**Patient**: Metformin, diuretics, and Lipitor.

**Doctor**: Do you have any allergies to medications or anything else?

**Patient**: No

**Doctor:** I would like to know more about your immediate family like your parents and your siblings. Are there any medical conditions in your family such as heart disease, diabetes, high blood pressure?

**Patient**: Yes. My parents have diabetes. My dad has a heart condition and I have a sister who also suffers from high blood pressure.

**Doctor**: I would like to know if you smoke.

**Patient**: No.

**Doctor**: Do you drink alcohol?

**Patient**: Yes, on some special occasions.

**Doctor**: Now I would like to examine you. First, I'm going to listen to your heart and then your lungs. Please take a deep breath every time you feel my stethoscope.

**Doctor**: Well Carmen, this is what we will do: since you have a high fever, cough, fatigue and loss of smell and taste, it is very likely that you have the COVID virus. We will test you for COVID. It usually takes a few hours to know the results. Someone from our hospital will call you when we have the result.

Your history and exam are reassuring, and the oxygen level in your blood is good. I think it is safe to discharge you. If you experience fever or headaches, you can take Motrin. If your symptoms worsen or you feel short of breath without exerting yourself, please return to the emergency room. What questions do you have?

**Patient**: Do you think my husband has the COVID virus too?

**Doctor**: It is possible. He should take a COVID test too. If he is negative, you should avoid direct contact as much as possible. You should wear facemasks while at home, wash your hands frequently, and clean all frequently touched objects well. Please wait here for the nurse who will provide you with the COVID test and give you your discharge papers.

**Doctor**: I hope you get better.

**Patient**: Thank you very much doctor!

**Spanish Script: Case 1**

**Doctor:** (toca la puerta)

**Paciente:** Adelante

**Doctor:** Hola Carmen, Soy la doctora ________, quien la atenderá en el día de hoy. Cuénteme, ¿cuál es el motivo de su visita?

**Paciente:** Doctora he estado con fiebre, tos y muy cansada estos días.

**Doctor:** ay, siento mucho que esté pasando por esta situación. Espero poder ayudarla.

Dígame, ¿Cuándo le empezaron los síntomas?

**Paciente**: hace tres días me empezó una tos seca y tomé anti-alérgicos porque pensé que tenía alergias. Pero luego empecé a sentirme muy cansada y con fiebre.

**Doctor:** ¿Cuándo fue la primera vez que notó la fiebre?

**Paciente**: hace dos días cuando mi esposo me tomó la temperatura y estaba en 102.5 grados.

**Doctor:** ¿Y qué hizo cuando se dio cuenta que tenía fiebre?

**Paciente:** tomé motrín y me acosté a dormir.

**Doctor:** ¿Sintió alguna mejoría con el motrín?

**Paciente:** Si, la fiebre se me bajó por unas horas pero al día siguiente volví a sentirme con fiebre. Además de la tos y el cansancio que no desaparecen.

**Doctor:** ¿Qué usted cree que le pasa?

**Paciente:** en verdad no lo sé. Estoy confundida con los síntomas de alergias, resfriado, la gripe y el virus del COVID-19. Doctora, ¿me podría explicar la diferencia?

**Doctor:** Esa es una muy buena pregunta Carmen. Las alergias causan picazón en los ojos o en la piel sin fiebre o contagio. Un resfriado común puede causar dolor de garganta, moqueo y tos. La gripe o influenza y el coronavirus son muy similares y pueden ser muy difícil distinguir basado solo en los síntomas.

¿Cree usted que ha estado expuesta a personas que tienen el COVID-19?

**Paciente:** En verdad no sé. Siempre trato de usar la mascarilla que me cubra la boca y la naríz. No he viajado ni he estado reuniéndome con muchas personas. Pero mi esposo y yo trabajamos y estamos expuestos. ¿Doctora usted cree que yo podría tener COVID?

**Doctor:** Es posible que sí por los síntomas que presenta. Pero primero debo hacerle más preguntas y luego debemos hacer los exámenes de detección para darle una respuesta definitiva. ¿Está bien?

**Paciente:** Si, me parece bien.

**Doctor:** Aparte de usted y su esposo, ¿vive alguien más en su casa?

**Paciente:** No. Solo somos él y yo.

**Doctor:** Y ¿su esposo también tiene los mismos síntomas que usted?

**Paciente:** No. Él solo se queja de que no puede oler o saborear la comida desde hace algunos dias.

**Doctor:** La pérdida del olfato y del gusto pueden ser causados por el COVID. Puede ser que usted o su esposo se hayan expuesto al virus sin saberlo. Al igual que su esposo, ¿ha experimentado usted la pérdida del olfato y/o del gusto?

**Paciente:** Si

**Doctor:** ¿Cuándo lo notó?

**Paciente:** He estado así hace algunos días pero no le presté mucha atención. Creo que desde hace tres días.

**Doctor:** ¿En qué trabaja?

**Paciente:** Trabajo cuidando niños.

**Doctor:** Ahora le quiero hacer otras preguntas más específicas para tener una mejor idea de lo que está pasando.

**Paciente:** Ok

**Doctor:** ¿Ha tenido dolor de cabeza?

**Paciente:** No

**Doctor:** ¿Ha tenido congestión nasal?

**Paciente:** No

**Doctor:** ¿Ha tenido dolor de garganta?

**Paciente:** Si. Creo que el dolor de garganta me empezó al mismo tiempo.

**Doctor:** Ahora mismo, ¿siente dificultad para respirar?

**Paciente:** No.

**Doctor:** Y cuando camina, ¿siente que le falta el aire?

**Paciente:** No.

**Doctor:** ¿Ha tenido dolor en el pecho?

**Paciente:** No

**Doctor:** ¿Ha tenido dolor abdominal, vómito o diarrea?

**Paciente:** No

**Doctor:** Ahora quisiera saber más sobre sus antecedentes médicos. ¿Qué condiciones médicas tiene?

**Paciente:** Sufro de diabetes tipo 2, presión arterial alta y colesterol.

**Doctor:** ¿Qué medicamentos toma?

**Paciente**: Metformin, diuréticos y Lipitor.

**Doctor:** ¿Tiene alguna alergia a medicamentos o a alguna otra cosa?

**Paciente:** No

**Doctor:** Me gustaría saber más sobre su familia inmediata como sus padres y sus hermanos/as.

¿Hay alguna condición médica en su familia? Como por ejemplo enfermedades del corazón, diabetes, presión arterial alta.

**Paciente**: Si. Mis padres tienen diabetes. Mi papá sufre del corazón y tengo una hermana que también sufre de presión alta.

**Doctor:** Me gustaría saber si usted fuma.

**Paciente:** No.

**Doctor:** ¿Toma alcohol?

**Paciente:** Si, en algunas ocasiones especiales.

**Doctor:** Ahora me gustaría examinarla. Primero le voy a escuchar el corazón y luego los pulmones.

**Examen físico: (Doctor)** Por favor respire profundo cada vez que sienta mi estetoscopio.

**Doctor:** Bueno Carmen esto es lo que haremos: ya que usted presenta con fiebre alta, tos, cansancio y falta de olfato y gusto, es muy probable que tenga el virus del COVID. Le haremos la prueba de detección del COVID. Por lo general tarda unas horas para saber los resultados. Alguien de nuestro hospital le llamará cuando tengamos el resultado.

Su historia y examen son tranquilizadores, y el nivel de oxígeno en su sangre es bueno. Creo que es seguro que se vaya a su casa. Si siente fiebre o dolor de cabeza puede tomar motrín. Si sus síntomas empeoran o usted siente dificultad al respirar sin poner esfuerzo, por favor regrese a la sala de emergencias.

¿Qué preguntas tiene?

**Paciente**: ¿Cree que mi esposo tiene el virus del COVID también?

**Doctor:** Es posible. El debe hacerse una prueba de COVID también. Si él está negativo, deben evitar contacto directo lo más posible. Deben siempre ponerse la mascarilla aun estando en la casa, lavarse las manos con frecuencia y limpiar bien todos los objetos frecuentemente tocados.

**Doctor:** Por favor espere aquí al enfermero quien le suministrará el exámen de COVID y le dará sus papeles de alta.

**Doctor:** Espero que se mejore.

**Paciente:** ¡Muchas gracias doctora!

**English Script: Case 2**

**Doctor**: (knocks on the door)

**Patient**: Go ahead

**Doctor**: Hello Carmen, I am doctor _________, I’ll be seeing you today. Tell me, what is the reason for your visit?

**Patient**: Doctor I have had a fever, and a cough and I’ve been very tired these past few days.

**Doctor**: Oh, I am very sorry that you are going through this. I hope I can help you. Tell me, when did your symptoms start?

**Patient**: Three days ago I had a dry cough and I took allergy medicine because I thought I had allergies. But then I started to feel very tired and feverish.

**Doctor**: When was the first time you noticed the fever?

**Patient**: Two days ago when my husband took my temperature and it was 102.5 degrees.

**Doctor**: And what did you do when you realized you had a fever?

**Patient**: I took Motrin and laid down to sleep.

**Doctor**: Did you feel any improvement with Motrin?

**Patient**: Yes, my fever went down for a few hours, but the next day I felt a fever again. Along with the cough and fatigue that do not go away.

**Doctor**: What do you think is wrong?

**Patient**: I really don't know. I am confused with the symptoms of allergies, the common cold, the flu, and the COVID-19 virus. Doctor, could you explain the difference?

**Doctor**: That’s a very good question Carmen. Allergies do not produce fever, are not contagious, and cause itchy eyes or itchy skin. The common cold may cause sore throat, runny nose, and cough. The flu and COVID-19 present similarly and it can be very difficult to differentiate them based just on symptoms.

Do you think you have been exposed to people who have COVID-19?

**Patient**: I really don't know. I always try to wear a mask that covers my mouth and nose. I have not traveled or been meeting with a lot of people. But my husband and I work and we are exposed.

Doctor, do you think I could have COVID?

**Doctor**: It is possible given your symptoms. But first I need to ask you more questions and then we need to do the screening tests to give you a definitive answer. Is that okay?

**Patient:** Yes, that's fine with me.

**Doctor**: Other than you and your husband, does anyone else live in your house?

**Patient**: No. It's just me and him.

**Doctor**: And does your husband also have the same symptoms as you?

**Patient**: No. He just complains that he cannot smell or taste food for a few days.

**Doctor**: Loss of smell and taste can be caused by COVID. It may be that you or your husband have been exposed to the virus without knowing it. Like your husband, have you experienced loss of smell and / or taste?

**Patient**: Yes

**Doctor**: When did you notice it?

**Patient**: I've been like this for a few days, but I didn't pay much attention to it. I think three days ago.
Doctor: What do you do for work?

**Patient**: I work in childcare.

**Doctor:** Now I want to ask you other more specific questions to get a better idea of what is going on.

**Patient:** Ok

**Doctor:** Have you had a headache?

**Patient:** No

**Doctor:** Have you had a stuffy nose?

**Patient**: Yes.

**Doctor**: Have you had a sore throat?

**Patient**: Yes. I think the sore throat started at the same time.

**Doctor**: Right now, do you feel short of breath?

**Patient**: Yes.

**Doctor**: And when you walk, do you feel short of breath?

**Patient**: Yes.

**Doctor:** Have you had chest pain?

**Patient**: No

**Doctor**: Have you had abdominal pain, vomiting, or diarrhea?

**Patient**: No

**Doctor:** Now I would like to know more about your medical history. What medical conditions do you have?

**Patient**: I have type 2 diabetes, high blood pressure and high cholesterol.

**Doctor**: What medicines do you take?

**Patient**: Metformin, diuretics and Lipitor.

**Doctor**: Do you have any allergies to medications or anything else?

**Patient**: No

**Doctor:** I would like to know more about your immediate family like your parents and your siblings. Are there any medical conditions in your family such as heart disease, diabetes, high blood pressure?

**Patient**: Yes. My parents have diabetes. My dad has a heart condition and I have a sister who also suffers from high blood pressure.

**Doctor**: I would like to know if you smoke.

**Patient**: No.

**Doctor**: Do you drink alcohol?

**Patient**: Yes, on some special occasions.

**Doctor**: Now I would like to examine you. First, I'm going to listen to your heart and then your lungs. Please take a deep breath every time you feel my stethoscope.

**Doctor**: Well Carmen, this is what we will do: since you have a high fever, cough, fatigue, lack of smell and taste, and difficulty breathing, it is very likely that you have the COVID virus. We will test you for COVID. It usually takes a few hours to know the results. The oxygen level in your blood is 88%. Normal is more than 94%. We're going to give you oxygen through a nasal cannula and admit you to the hospital. We are also going to start a course of steroids to lower the inflammation in your lungs. One of my colleagues from the medical service is coming to talk more about your admission. What questions do you have?

**Patient**: Do you think my husband has the COVID virus too?

**Doctor**: It is possible. He should take a COVID test too. If he is negative, you should avoid direct contact as much as possible. You should wear facemasks while at home, wash your hands frequently, and clean all frequently touched objects well.

Please wait here for my colleague who will begin the admission process. I hope you get well soon!

**Patient**: Thank you very much doctor!

**Spanish Script: Case 2**

**Doctor:** toca la puerta.

**Paciente:** Adelante

**Doctor:** Hola Carmen, Soy la doctora _________, quien la atenderá en el día de hoy. Cuénteme, ¿cuál es el motivo de su visita?

**Paciente:** doctora he estado con fiebre, tos y muy cansada estos días.

**Doctor:** ay, siento mucho que esté pasando por esta situación. Espero poder ayudarla.

Dígame, ¿Cuándo le empezaron los síntomas?

**Paciente**: hace tres días me empezó una tos seca y tomé anti-alérgicos porque pensé que tenía alergias. Pero luego empecé a sentirme muy cansada y con fiebre.

**Doctor:** ¿Cuándo fue la primera vez que notó la fiebre?

**Paciente**: hace dos días cuando mi esposo me tomó la temperatura y estaba en 102.5 grados.

**Doctor:** ¿Y qué hizo cuando se dio cuenta que tenía fiebre?

**Paciente:** tomé motrín y me acosté a dormir.

**Doctor:** ¿Sintió alguna mejoría con el motrín?

**Paciente:** Si, la fiebre se me bajó por unas horas pero al día siguiente volví a sentirme con fiebre. Además de la tos y el cansancio que no desaparecen.

**Doctor:** ¿Qué usted cree que le pasa?

**Paciente:** en verdad no lo sé. Estoy confundida con los síntomas de alergias, resfriado, la gripe y el virus del COVID-19. Doctora, ¿me podría explicar la diferencia?

**Doctor:** Esa es una muy buena pregunta Carmen. Las alergias causan picazón en los ojos o en la piel sin fiebre o contagio. Un resfriado común puede causar dolor de garganta, moqueo y tos. La gripe o influenza y el coronavirus son muy similares y pueden ser muy difícil distinguir basado solo en los síntomas.

¿Cree usted que ha estado expuesta a personas que tienen el COVID-19?

**Paciente:** En verdad no sé. Siempre trato de usar la mascarilla que me cubra la boca y la naríz. No he viajado ni he estado reuniéndome con muchas personas. Pero mi esposo y yo trabajamos y estamos expuestos. ¿Doctora usted cree que yo podría tener COVID?

**Doctor:** Es posible que sí por los síntomas que presenta. Pero primero debo hacerle más preguntas y luego debemos hacer los exámenes de detección para darle una respuesta definitiva. ¿Está bien?

**Paciente:** Si, me parece bien.

**Doctor:** Aparte de usted y su esposo, ¿vive alguien más en su casa?

**Paciente:** No. Solo somos él y yo.

**Doctor:** Y ¿su esposo también tiene los mismos síntomas que usted?

**Paciente:** No. Él solo se queja de que no puede oler o saborear la comida desde hace algunos dias.

**Doctor:** La pérdida del olfato y del gusto pueden ser causados por el COVID. Puede ser que usted o su esposo se hayan expuesto al virus sin saberlo. Al igual que su esposo, ¿ha experimentado usted la pérdida del olfato y/o del gusto?

**Paciente:** Si

**Doctor:** ¿Cuándo lo notó?

**Paciente:** He estado así hace algunos días pero no le presté mucha atención. Creo que desde hace tres días.

**Doctor:** ¿En qué trabaja?

**Paciente:** Trabajo cuidando niños.

**Doctor:** Ahora le quiero hacer otras preguntas más específicas para tener una mejor idea de lo que está pasando.

**Paciente:** Ok

**Doctor:** ¿Ha tenido dolor de cabeza?

**Paciente:** No

**Doctor:** ¿Ha tenido congestión nasal?

**Paciente:** Si

**Doctor:** ¿Ha tenido dolor de garganta?

**Paciente:** Si. Creo que el dolor de garganta me empezó al mismo tiempo.

**Doctor:** Ahora mismo, ¿siente dificultad para respirar?

**Paciente:** Si

**Doctor:** Y cuando camina, ¿siente que le falta el aire?

**Paciente:** Si

**Doctor:** ¿Ha tenido dolor en el pecho?

**Paciente:** No

**Doctor:** ¿Ha tenido dolor abdominal, vómito o diarrea?

**Paciente:** No

**Doctor:** Ahora quisiera saber más sobre sus antecedentes médicos. ¿Qué condiciones médicas tiene?

**Paciente:** Sufro de diabetes tipo 2, presión arterial alta y colesterol.

**Doctor:** ¿Qué medicamentos toma?

**Paciente**: Metformin, diuréticos y Lipitor.

**Doctor:** ¿Tiene alguna alergia a medicamentos o a alguna otra cosa?

**Paciente:** No

**Doctor:** Me gustaría saber más sobre su familia inmediata como sus padres y sus hermanos/as.

Hay alguna condición médica en su familia como por ejemplo enfermedades del corazón, diabetes, presión arterial alta?

**Paciente**: Si. Mis padres tienen diabetes. Mi papá sufre del corazón y tengo una hermana que también sufre de presión alta.

**Doctor:** Me gustaría saber si usted fuma.

**Paciente:** No.

**Doctor:** ¿Toma alcohol?

**Paciente:** Si, en algunas ocasiones especiales.

**Doctor:** Ahora me gustaría examinarla. Primero le voy a escuchar el corazón y luego los pulmones.

**Examen físico: (Doctor)** Por favor respire profundo cada vez que sienta mi estetoscopio.

**Doctor:** Bueno Carmen esto es lo que haremos: ya que usted presenta con fiebre alta,

tos, cansancio, falta de olfato y gusto, y dificultad para respirar es muy probable que tenga el virus del COVID. Le haremos la prueba de detección del COVID. Por lo general tarda unas horas para saber los resultados. El nivel de oxígeno en su sangre es 88%. Lo normal es más de 94%. Vamos a suministrarle oxígeno por una cánula nasal y admitirla al hospital. También vamos a empezar un curso de esteroides para bajar la inflamación en los pulmones. Uno de mis colegas del servicio de medicina vendrá para hablar más sobre su

admisión.

¿Qué preguntas tiene?

**Paciente**: ¿Cree que mi esposo tiene el virus del COVID también?

**Doctor:** Es posible. El debe tomar una prueba de COVID también. Si él no está positivo, deben evitar contacto directo lo más posible. Deben ponerse la mascarilla aun estando en casa, lavarse las manos con frecuencia y limpiar bien todos los muebles y objetos frecuentemente tocados.

Espere aquí a mi colega quien hará el proceso para admitirla. ¡Espero que se mejore pronto!

**Paciente**: ¡Muchas gracias doctora!
