# Supplementary material for: COVID-19 Education for Health Professionals Caring for Spanish-Speaking Patients
Source: MedEdPORTAL. 2022 Apr 12;18:11240. doi: 10.15766/mep_2374-8265.11240 (PMC9001760; doi:10.15766/mep_2374-8265.11240)
Supplement: Supplementary file 1 — Facilitator Guide.docxCOVID-19 Presentation.pptxSpanish Clinical Encounter for Case 1.mp4English Clinical Encounter for Case 1.mp4Spanish Clinical Encounter for Case 2.mp4English Clinical Encounter for Case 2.mp4English and Spanish Scripts for Cases 1 & 2.docxPostworkshop Evaluation.docx [file mep_2374-8265.11240-s001.zip › H. Postworkshop Evaluation.docx]

**COVID-19 Education for Health Professionals Caring for Spanish-Speaking Patients**

**Post-Test**

**Part I. Self-Assessment**

| **Please rate how much CONFIDENCE do you have in your ability to…** | **No Confidence 0** | **1** | **2** | **3** | **Complete Confidence 4** |
| --- | --- | --- | --- | --- | --- |
| Obj 1: Discuss the diagnosis, symptoms, treatment and vaccines of COVID-19 with a Spanish speaking patient | 0 | 1 | 2 | 3 | 4 |
| Obj 2: Compare the epidemiology of COVID-19 in regards to race and ethnicity | 0 | 1 | 2 | 3 | 4 |
| Obj 3: Discuss plan to discharge home versus admission to the hospital with a Spanish Speaking patient. | 0 | 1 | 2 | 3 | 4 |

**Part II. Objective Assessment**

**Please Choose the Best Answer:**

1. Hispanics and Latinos are ___ X more likely to be hospitalized due to COVID-19

a. 2

b. 1

c. 6

d. 4

2. In Spanish, what is the best way to prevent COVID-19?

a. Remdesivir

b. Motrin y Tylenol

c. Una mascarilla/Un cubrebocas

d. Caldo de pollo

3. In Spanish, what are the common side effects of the COVID-19 vaccines:

a. Fiebre baja, dolor muscular, cansancio

b. Dolor de pecho

c. Tos y dolor de garganta

d. Enfermedad renal crónica

**Part III. Comments**

**Please answer the following questions:**

1. What did you like about this workshop?

2. What suggestions do you have to improve this workshop?
