# Supplementary material for: COVID-19 Education for Health Professionals Caring for Spanish-Speaking Patients
Source: MedEdPORTAL. 2022 Apr 12;18:11240. doi: 10.15766/mep_2374-8265.11240 (PMC9001760; doi:10.15766/mep_2374-8265.11240)
Supplement: Supplementary file 1 — Facilitator Guide.docxCOVID-19 Presentation.pptxSpanish Clinical Encounter for Case 1.mp4English Clinical Encounter for Case 1.mp4Spanish Clinical Encounter for Case 2.mp4English Clinical Encounter for Case 2.mp4English and Spanish Scripts for Cases 1 & 2.docxPostworkshop Evaluation.docx [file mep_2374-8265.11240-s001.zip › A. Facilitator Guide.docx]

**COVID-19 Education for Health Professionals Caring for Spanish-Speaking Patients**

**Facilitator Guide**

**Overall Goals**

The goals of this module are to help health professionals and trainees communicate effectively and efficiently when caring for Spanish-speaking patients, specifically when discussing signs, symptoms, treatment options, disease epidemiology, and vaccination options related to COVID-19.

**Workshop Objectives**

1. Discuss the symptoms, diagnosis, treatment, and vaccines associated with COVID-19 with a Spanish speaking patient;

2. Compare the epidemiology of COVID-19 regarding race and ethnicity;

3. Discuss plan to discharge home vs admission to the hospital with a Spanish Speaking patient.

**Workshop Handouts and Materials:**

COVID-19 workshop PPT slide set

Spanish and English scripts and videos

Computer setup

Zoom room set up with breakout rooms

Postworkshop evaluation form

Suggested additional readings:

World Health Organization. (n.d.). *Coronavirus*. World Health Organization. Retrieved January 17, 2022, from https://www.who.int/health-topics/coronavirus#tab=tab_1

*COVID-19: Epidemiology, virology, and prevention*. UpToDate. (n.d.). Retrieved January 17, 2022, from https://www.uptodate.com/contents/covid-19-epidemiology-virology-and-prevention?search=Coronavirus%3A+Epidemiology%2C+virology+and+prevention.&source=search_result&selectedTitle=2~150&usage_type=default&display_rank=1

**Suggested Agenda and Timeline:**

PowerPoint presentation: 15 minutes

*Videos showcase: 5-10 minutes

Practice scripts: 30 minutes

Feedback: 10 minutes

Post-workshop evaluation: 2-3 minutes

*Timeline does not include the showcase of the videos. These videos of the scripts are provided in English and Spanish as optional materials during the workshop for an enhanced auditory experience.

**For Facilitators that allot more time for the workshop, alternative information may be added to the presentation when discussing slides and/or practice with scripts may be in a small group format.

**Suggested Reading Material and Resources for Facilitators in Preparation for Workshop:**

- Management and clinical guidelines for COVID-19: https://www.cdc.gov/coronavirus/2019-ncov/hcp/clinical-guidance-management-patients.html
- Therapeutic options for COVID-19: https://www.cdc.gov/coronavirus/2019-ncov/hcp/therapeutic-options.html
- Race, ethnicity and COVID-19: https://www.cdc.gov/coronavirus/2019-ncov/covid-data/investigations-discovery/hospitalization-death-by-race-ethnicity.html
- Clinical management of COVID-19: https://www.who.int/publications/i/item/clinical-management-of-covid-19
- Social determinants of health:

https://www.cdc.gov/socialdeterminants/index.htm

**Slide Instructions:**

The number of facilitators will be dependent on availability. One facilitator is enough for the workshop as it was implemented. One to three facilitators is recommended.

**Slide 1: Title Slide**

Title Slide.

**Slide 2: Disclaimer/Descargo de Responsabilidad Slide**

**Slide 3: Presenters/presentadores slide**

Presenters should introduce themselves and their respective academic title as well as their respective institutions. They may also share their Hispanic, Latino or Spanish origin/identity.

Los presentadores deben presentarse a sí mismos y a su respectivo título académico, así como a sus respectivas instituciones. También pueden compartir su origen/identidad.

**Slide 4: Disclosure/divulgaciones slide**

Presenters should state if they have any disclosures.

Los presentadores deben indicar si tienen alguna divulgación.

**Slide 5: Objectives/objetivos slide**

Presenters should discuss the objectives of the workshop. By the end of the workshop, participants should be able to, in English and Spanish, be able to define COVID-19, discuss the epidemiology of COVID-19 and how it pertains to race and ethnicity, list symptoms of COVID-19, and describe the treatment and prevention of COVID-19.

Los presentadores deben discutir los objetivos del taller. Al final del taller, los participantes deberían poder, en inglés y español, definir COVID-19, discutir la epidemiología de COVID-19 y cómo se relaciona con la raza y la etnia, enumerar los síntomas de COVID-19 y describir el tratamiento de prevención de COVID-19.

**Slide 6: Definition/definición slide**

Presenters should briefly define COVID-19.

Los presentadores deben definir brevemente COVID-19.

**Slide 7: Cases by Race and Ethnicity**

Presenters should discuss the graphic from the CDC. The graphic shows that while 55.9% of COVID-19 cases are White, Non-Hispanic, an increasing number of cases are of Hispanic or Latino origin. Total cases counted were 15,115,825 cases. Race and ethnicity data was available for 7,764,698 cases or 51%.

Los presentadores deben discutir el gráfico de la CDC. El gráfico muestra que si bien el 55.9% de los casos de COVID-19 son blancos, no hispanos, un número creciente de casos son de origen Hispano o Latino. El total de casos contados fue de 15,115,825 casos. Los datos de raza y etnia estaban disponibles para 7,764,698 casos o 51%.

**Slide 8: Casos por Raza y Etnia**

Los presentadores deben discutir el gráfico de la CDC. El gráfico muestra que, si bien el 55.9% de los casos de COVID-19 son blancos, no hispanos, un número creciente de casos son de origen Hispano o Latino. El total de casos contados fue de 15,115,825 casos. Los datos de raza y etnia estaban disponibles para 7.764.698 casos o 51%.

**Slide 9: Deaths by Race and Ethnicity**

Presenters should note that Hispanic and Latino deaths account for 12.3% of all total deaths. Black, non-Hispanics make up 15.3%. This data was extrapolated from 249, 416 deaths. Race and ethnicity data was available for 194,620 deaths.

**Slide 10: Muertes por Raza y Etnia**

Los presentadores deben tener en cuenta que las muertes de Hispanos y Latinos representan el 12,3% del total de muertes. Los negros no hispanos representan el 15,3%. Estos datos fueron extrapolados de 249, 416 muertes. Los datos de raza y etnia estaban disponibles para 194,620 muertes.

**Slide 11: Cases, Hospitalizations, and Deaths by Race/Ethnicity**

Presenters should discuss that higher rates of death and hospitalizations in the Hispanic and Black communities may be due to inequities in social determinants of health. Higher rates of infection and death could be due to multigenerational and multifamily households, residing in closed living environments, frontline jobs, limited access to healthcare.

**Slide 12: Casos, Hospitalizaciones, y Muerte por Raza/Etnia**

Los presentadores deben discutir que las tasas más altas de muerte y hospitalizaciones en las comunidades hispanas y negras pueden deberse a desigualdades en los determinantes sociales de la salud. Las tasas más altas de infección y muerte podrían deberse a hogares multigeneracionales y multifamiliares, que residen en entornos de vida cerrados, trabajos de primera línea y acceso limitado a la atención médica.

**Slide 13: Symptoms/ Síntomas**

Presenters should briefly list the common symptoms of COVID-19 & state how these symptoms can be confused with the common cold or flu.

Spanish/Español:

Los presentadores deben nombrar los síntomas comunes de COVID-19 y explicar cómo los síntomas se parecen a un catarro o a la gripe.

**Slide 14: Risk Factors for Severe Disease/ Riesgo de Enfermedad Severa**

The purpose of this slide is to inform patients that certain risk factors exist that may make an infection with COVID-19 severe. Presenters may name a few examples of risk factors on this slide.

Spanish/Espanol:

El objetivo de esta diapositiva es para informar a los pacientes que existen ciertos factores de riesgo que pueden hacer que una infección por COVID-19 sea grave.

Presentadores pueden nombrar algunos de los riesgos en esta dispositiva.

**Slide 15: Treatment/ Tratamiento**

As of today, Remdesivir is the only FDA-approved medication to treat hospitalized patients with moderate to severe cases of COVID-19.

Motrin or Tylenol are available OTC (Over the counter) options to treat symptoms like pain or fever.

Presenters should inform of non-medication options like rest, hydration, and self-monitoring symptoms.

Spanish/Español:

A partir de hoy, Remdesivir es el único medicamento aprobado por el FDA para pacientes hospitalizados con casos de COVID-19 considerado moderados o severos.

Motrin o Tylenol están disponibles en la venta libre, sin receta, para ayudar con los síntomas de dolor o fiebre.

Los presentadores deben informar sobre opciones sin medicación como el descanso, hidratación y controlar los síntomas.

**Slide 16: Preventing Infection/ Cómo Prevenir la Infección**

There are many things patients can do to reduce their risk of exposure to COVID-19. Presenters should name a few options

Spanish/Español:

Hay varias cosas que los pacientes pueden hacer para disminuir el riesgo de contraer COVID-19. Los presentadores deben nombrar algunas de las opciones.

**Slide 17: The COVID Vaccine/ La Vacuna de COVID**

Presenters should transition into talking about the COVID Vaccine. The presenter should discuss that there are currently two vaccines that have been authorized by the FDA:

1. The Pfizer-BioNTech

2. Moderna

Emphasize that both are at least 94% effective and both cases require 2 doses

El presentador debe discutir que actualmente existen dos vacunas que han sido autorizadas por la administración de drogas y alimentos

1. The Pfizer-BioNTech

2. Moderna

Ambas son por lo menos 94% efectivas, y Ambas vacunas requieren de 2 dosis.

**Slide 18: How does it work? ¿Cómo funciona?**

The presenter should discuss the importance of vaccines in general, and delve into specifically how the COVID vaccine is working within our body

El presentador debe discutir la importancia de las vacunas en general, y comentar específicamente sobre cómo la vacuna del COVID funciona dentro de nuestro cuerpo

**Slide 19:** **Side Effects and Contraindications/ Efectos Secundarios y Contraindicaciones**

The presenter should discuss Common Side Effects and Contraindications to be aware of if getting the Vaccine. The most common side effects include muscle aches, fatigue, fevers, and soreness.

El presentador debe discutir los efectos secundarios y contraindicaciones importantes. Los efectos secundarios más comunes incluyen: dolor muscular, cansancio, fiebre y dolor en el sitio de la inyección.

**Slide 20:**

The facilitator should thank the audience for their time and attention and ask if there are any questions, comments, or concerns.

El presentador debe agradecer a la audiencia por su tiempo y su atención y también preguntar si hay dudas, comentarios o inquietudes.

**Video-Based Education and/or Role-Play Exercises**

After the presentation of the PPT slides, the facilitator(s) have the option of showing the videos in Spanish to allow for an auditory learning experience. The participants may be kept as one group or divided into multiple groups to role play using up to two different case scenarios. Participants should be given the English scripts and volunteers should be requested to serve as the patient or practitioner. The volunteers should be encouraged to communicate with each other in Spanish using the scripts in English. If the participants have difficulty in communicating the English script content in Spanish, the facilitator(s) should provide potential Spanish words and phrases to help the volunteers along.

**Feedback**

After practicing the scripts, participants will have the opportunity to provide verbal feedback to the facilitator(s) on their experience and how to improve the workshop.
