# Supplementary material for: COVID-19 Education for Health Professionals Caring for Spanish-Speaking Patients
Source: MedEdPORTAL. 2022 Apr 12;18:11240. doi: 10.15766/mep_2374-8265.11240 (PMC9001760; doi:10.15766/mep_2374-8265.11240)
Supplement: Supplementary file 1 — Facilitator Guide.docxCOVID-19 Presentation.pptxSpanish Clinical Encounter for Case 1.mp4English Clinical Encounter for Case 1.mp4Spanish Clinical Encounter for Case 2.mp4English Clinical Encounter for Case 2.mp4English and Spanish Scripts for Cases 1 & 2.docxPostworkshop Evaluation.docx [file mep_2374-8265.11240-s001.zip › B. COVID-19 Presentation.pptx]

## Slide 1
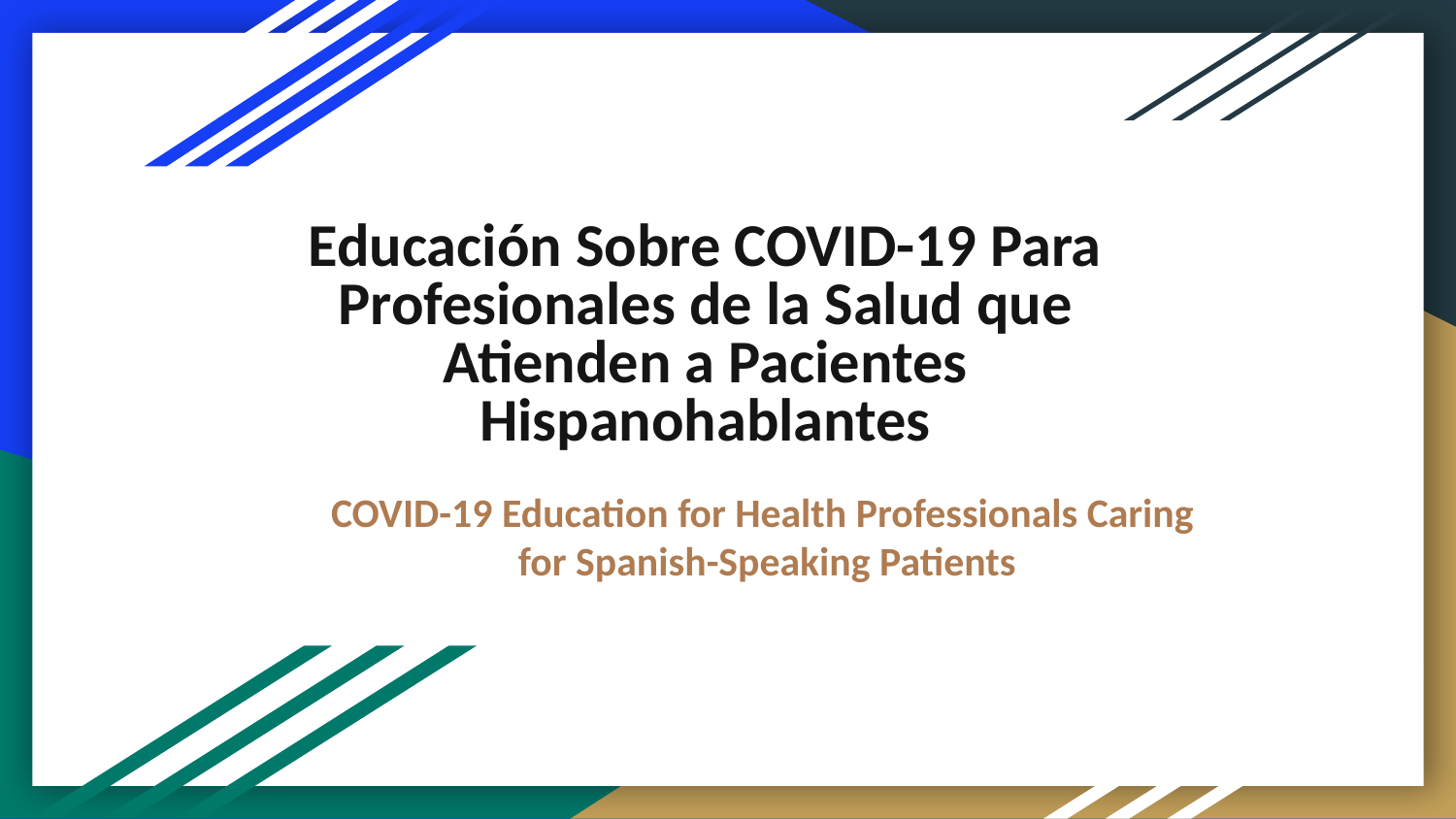

Educación Sobre COVID-19 Para Profesionales de la Salud que Atienden a Pacientes Hispanohablantes
# COVID-19 Education for Health Professionals Caring for Spanish-Speaking Patients

## Slide 2
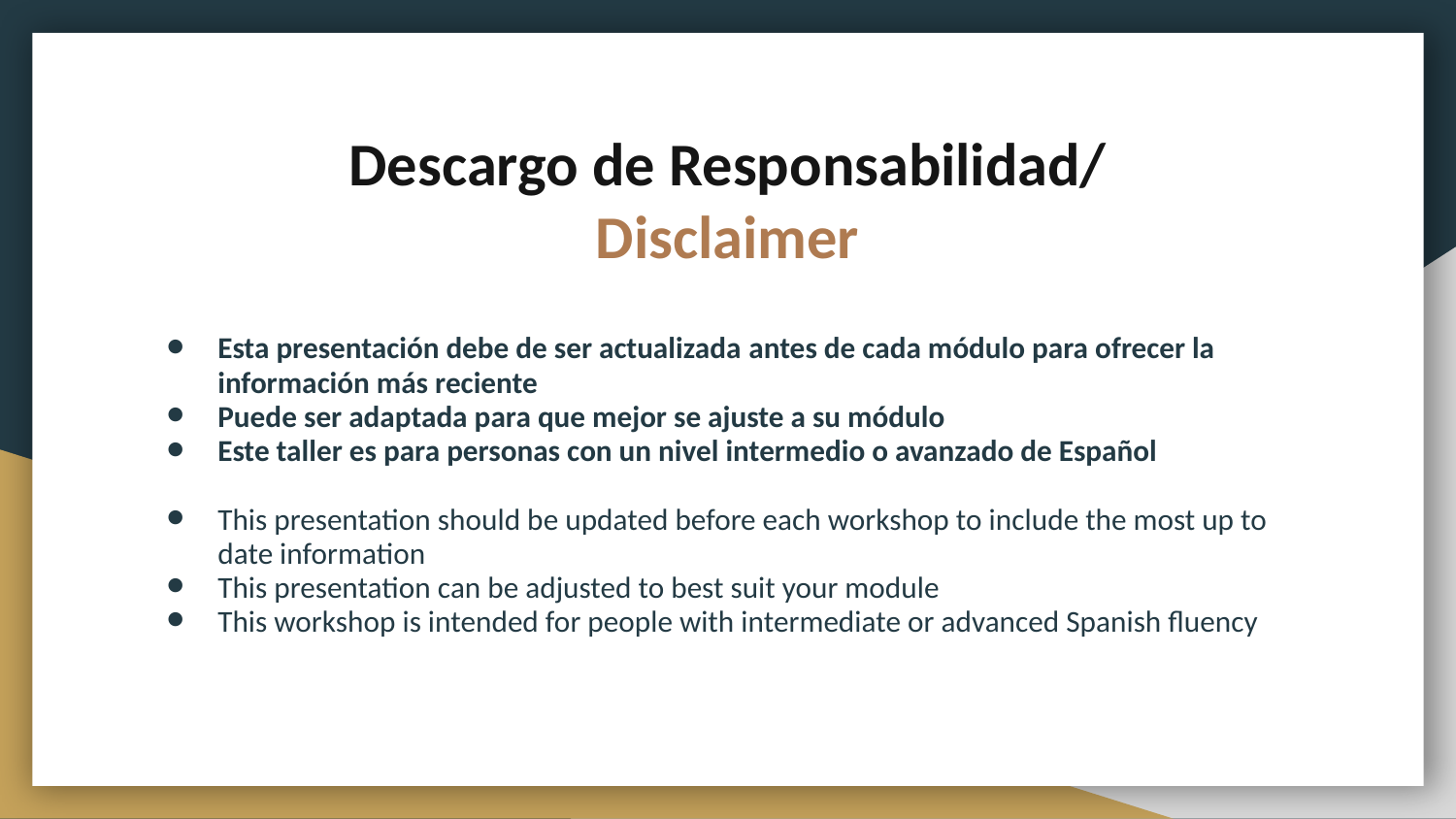

# Descargo de Responsabilidad/ Disclaimer
Esta presentación debe de ser actualizada antes de cada módulo para ofrecer la información más reciente
Puede ser adaptada para que mejor se ajuste a su módulo
Este taller es para personas con un nivel intermedio o avanzado de Español
This presentation should be updated before each workshop to include the most up to date information
This presentation can be adjusted to best suit your module
This workshop is intended for people with intermediate or advanced Spanish fluency

## Slide 3
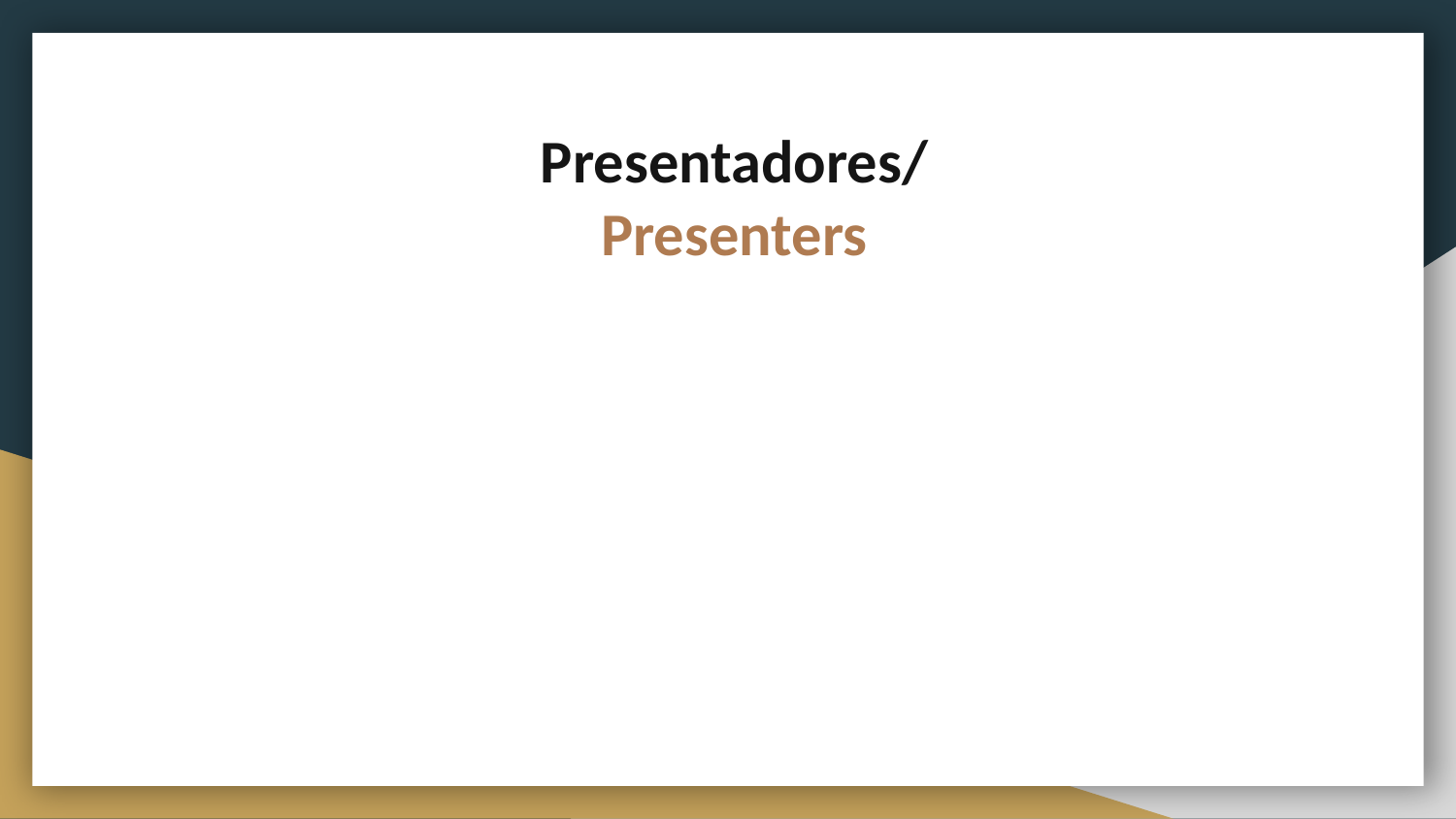

# Presentadores/
Presenters

## Slide 4
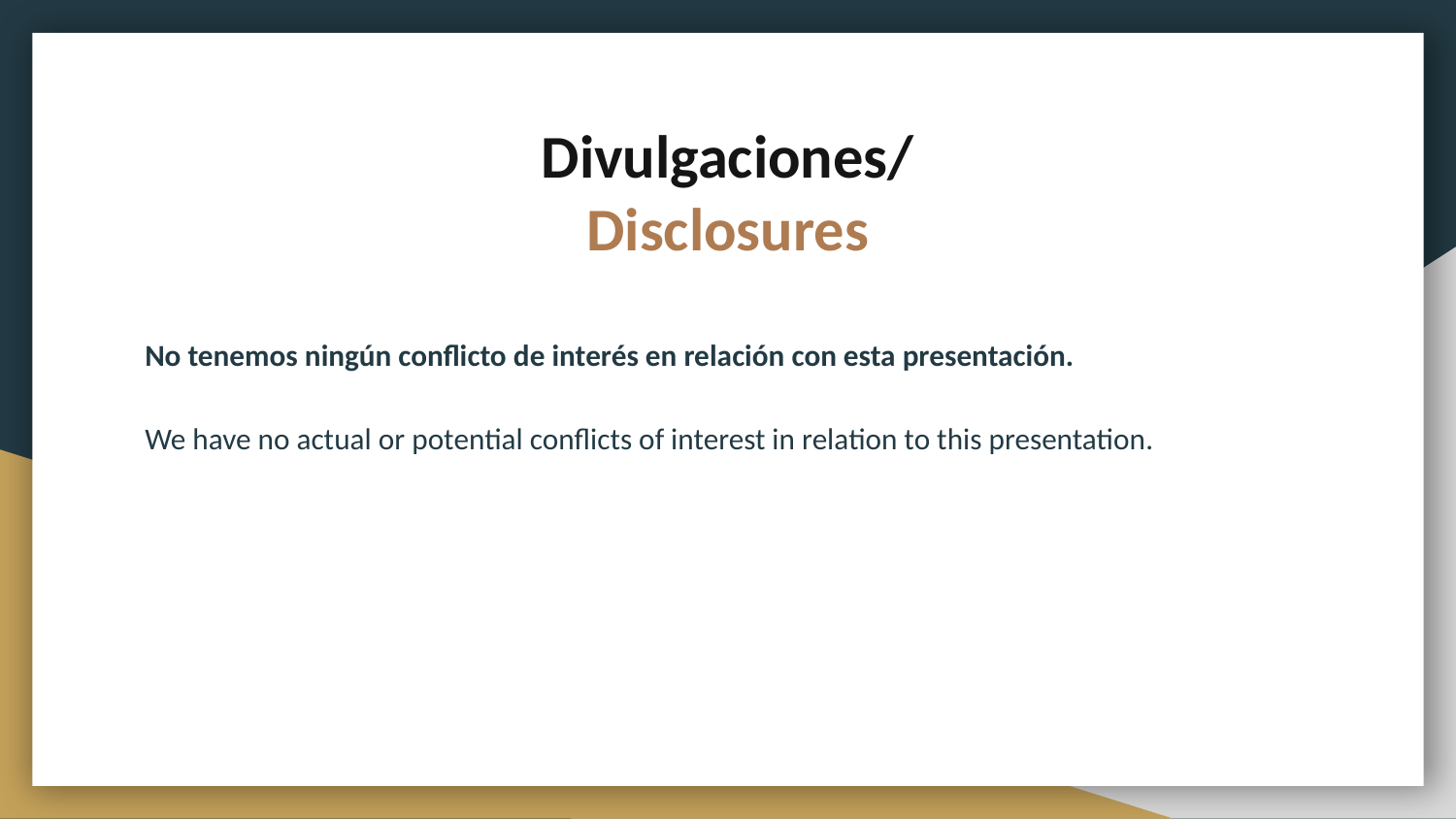

# Divulgaciones/
Disclosures
No tenemos ningún conflicto de interés en relación con esta presentación.
We have no actual or potential conflicts of interest in relation to this presentation.

## Slide 5
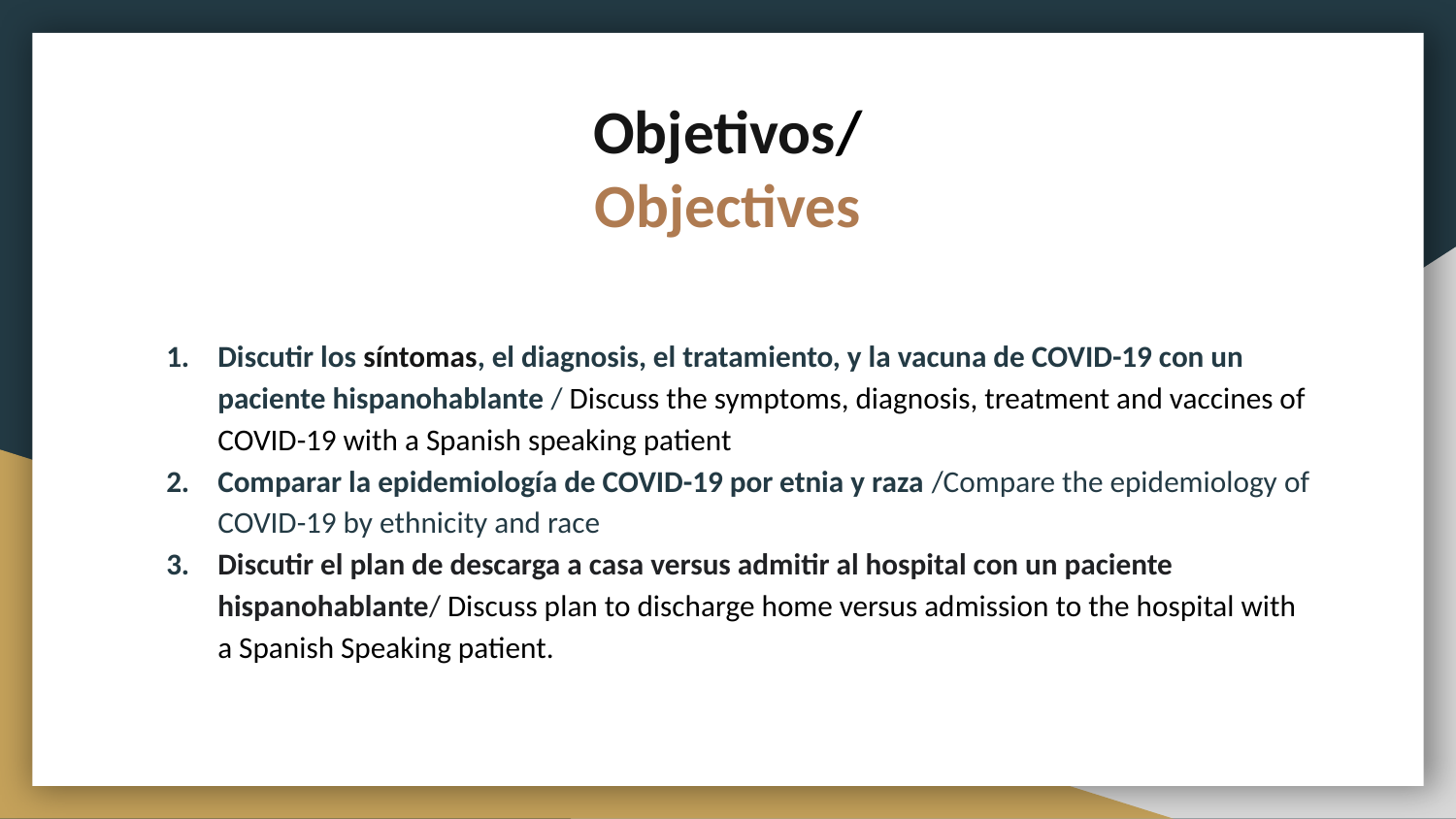

# Objetivos/
Objectives
Discutir los síntomas, el diagnosis, el tratamiento, y la vacuna de COVID-19 con un paciente hispanohablante / Discuss the symptoms, diagnosis, treatment and vaccines of COVID-19 with a Spanish speaking patient
Comparar la epidemiología de COVID-19 por etnia y raza /Compare the epidemiology of COVID-19 by ethnicity and race
Discutir el plan de descarga a casa versus admitir al hospital con un paciente hispanohablante/ Discuss plan to discharge home versus admission to the hospital with a Spanish Speaking patient.

## Slide 6
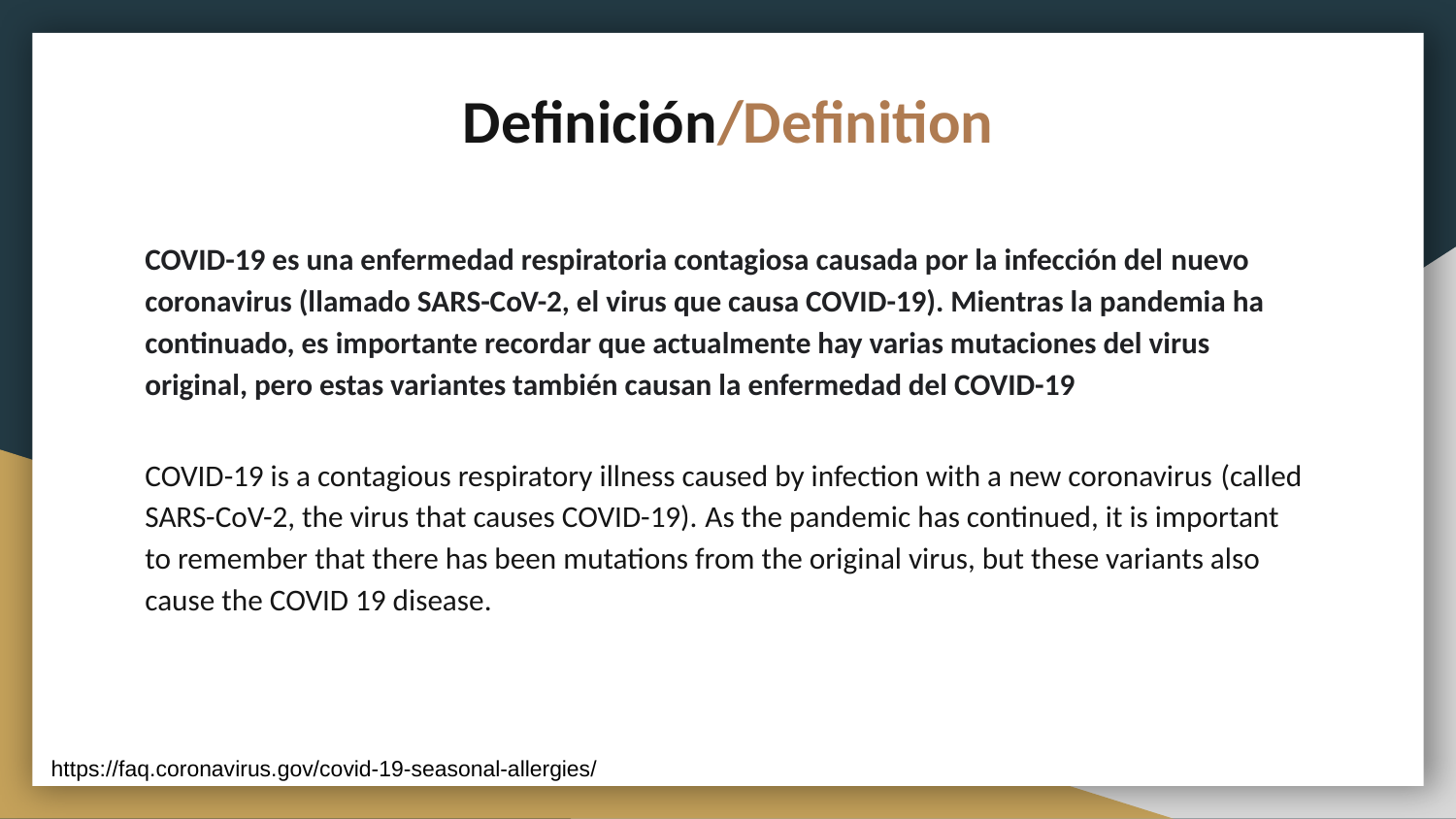

# Definición/Definition
COVID-19 es una enfermedad respiratoria contagiosa causada por la infección del nuevo coronavirus (llamado SARS-CoV-2, el virus que causa COVID-19). Mientras la pandemia ha continuado, es importante recordar que actualmente hay varias mutaciones del virus original, pero estas variantes también causan la enfermedad del COVID-19
COVID-19 is a contagious respiratory illness caused by infection with a new coronavirus (called SARS-CoV-2, the virus that causes COVID-19). As the pandemic has continued, it is important to remember that there has been mutations from the original virus, but these variants also cause the COVID 19 disease.
https://faq.coronavirus.gov/covid-19-seasonal-allergies/

## Slide 7
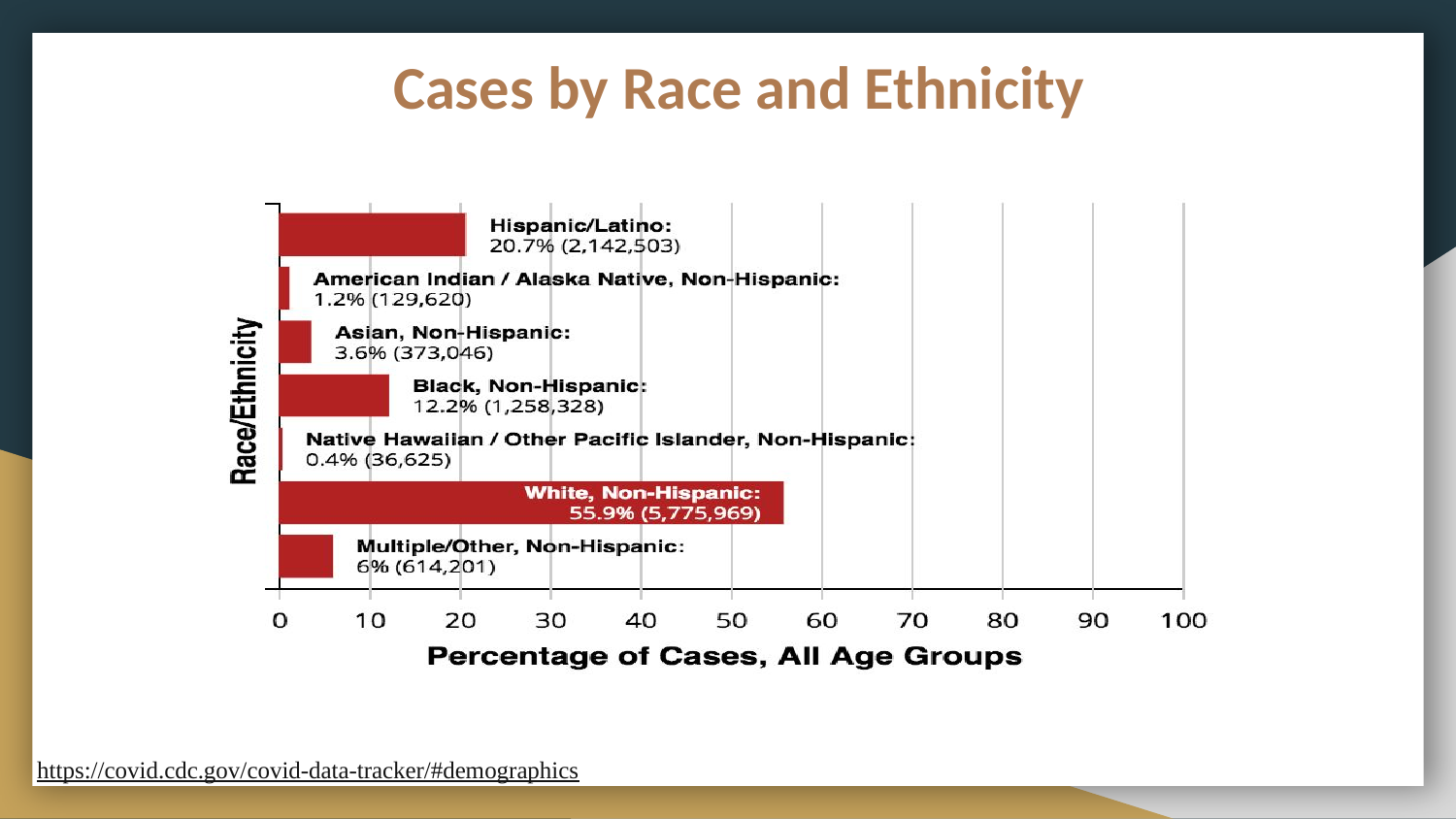

# Cases by Race and Ethnicity
https://covid.cdc.gov/covid-data-tracker/#demographics

## Slide 8
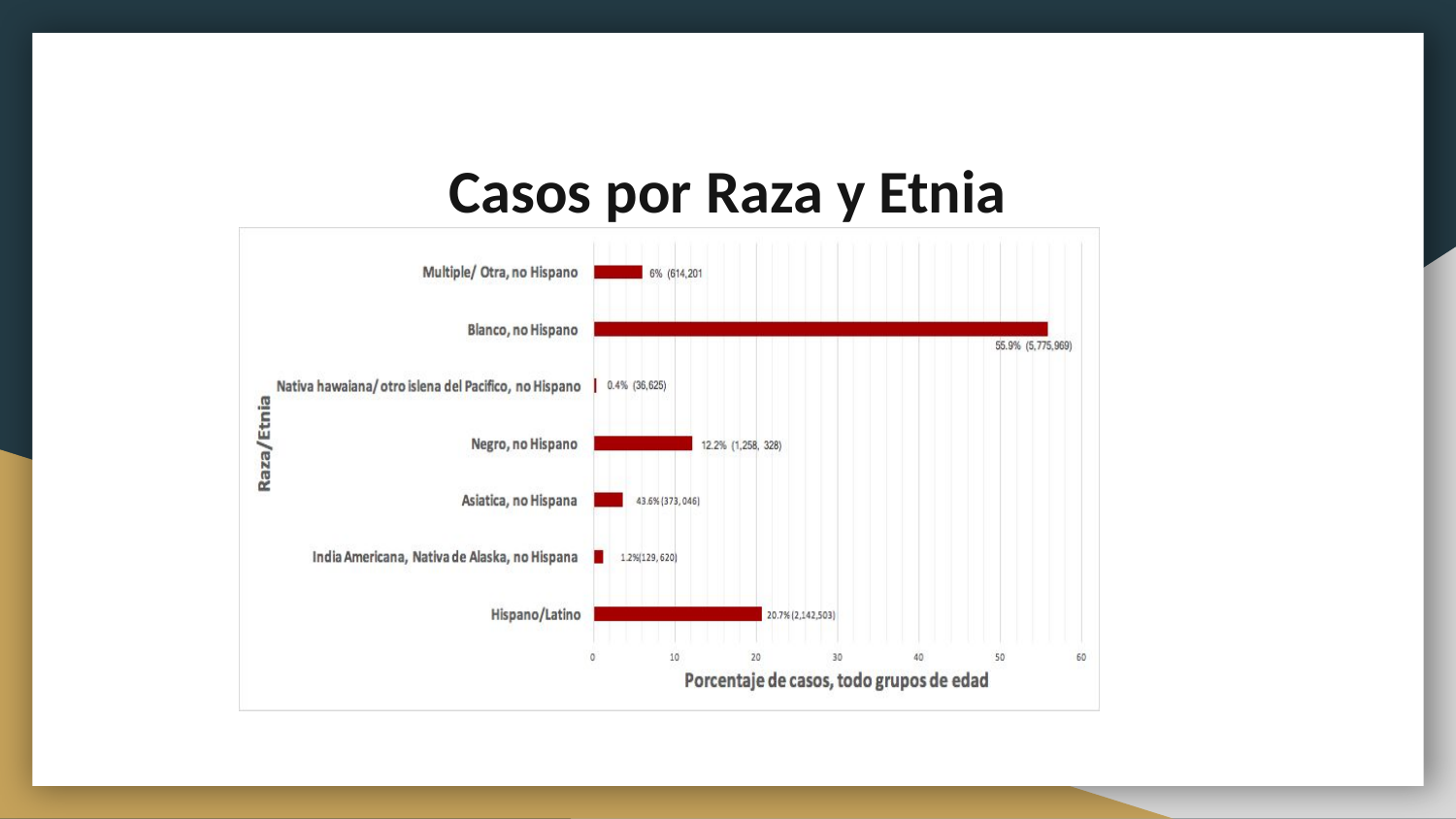

# Casos por Raza y Etnia

## Slide 9
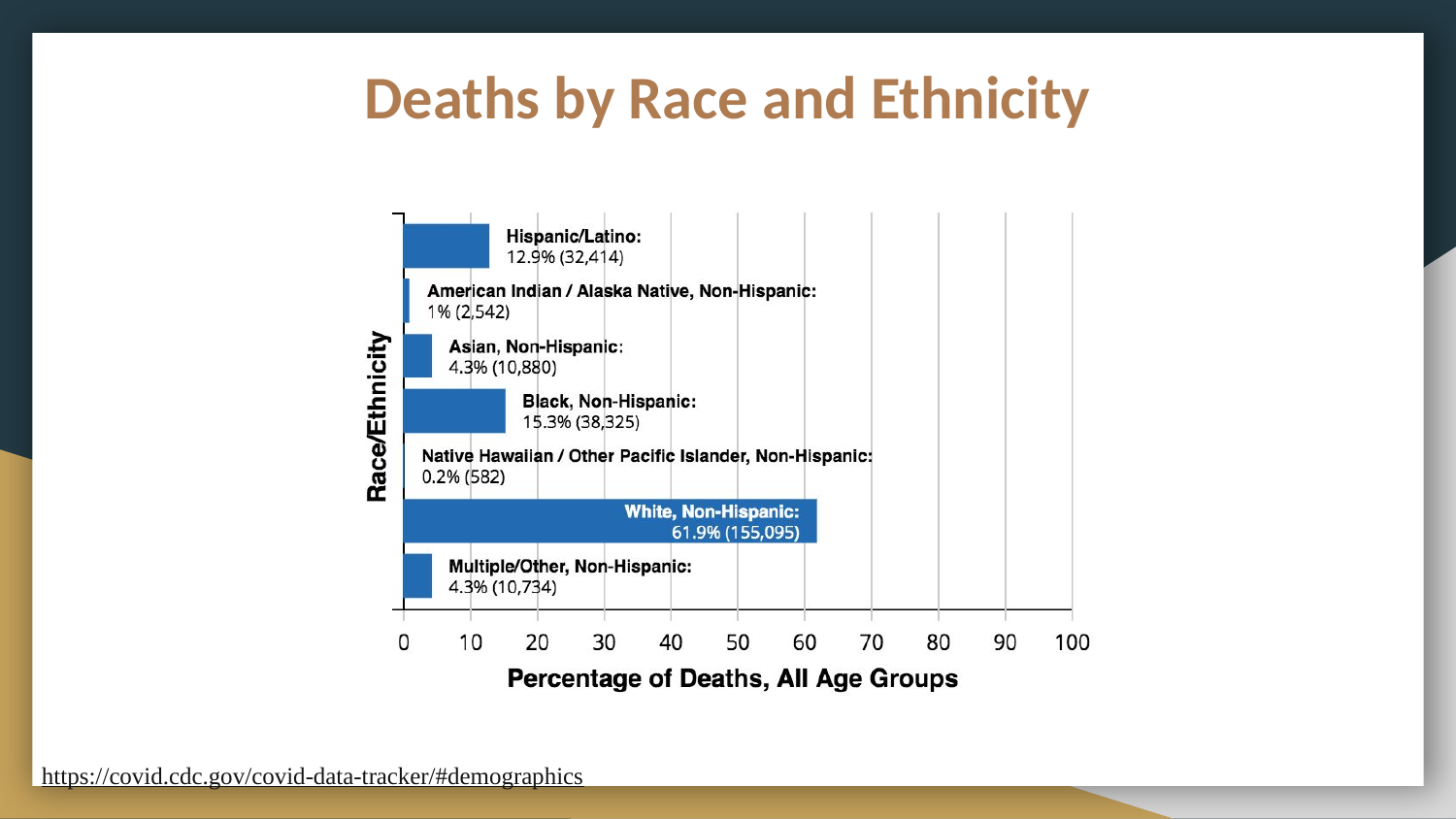

# Deaths by Race and Ethnicity
https://covid.cdc.gov/covid-data-tracker/#demographics

## Slide 10
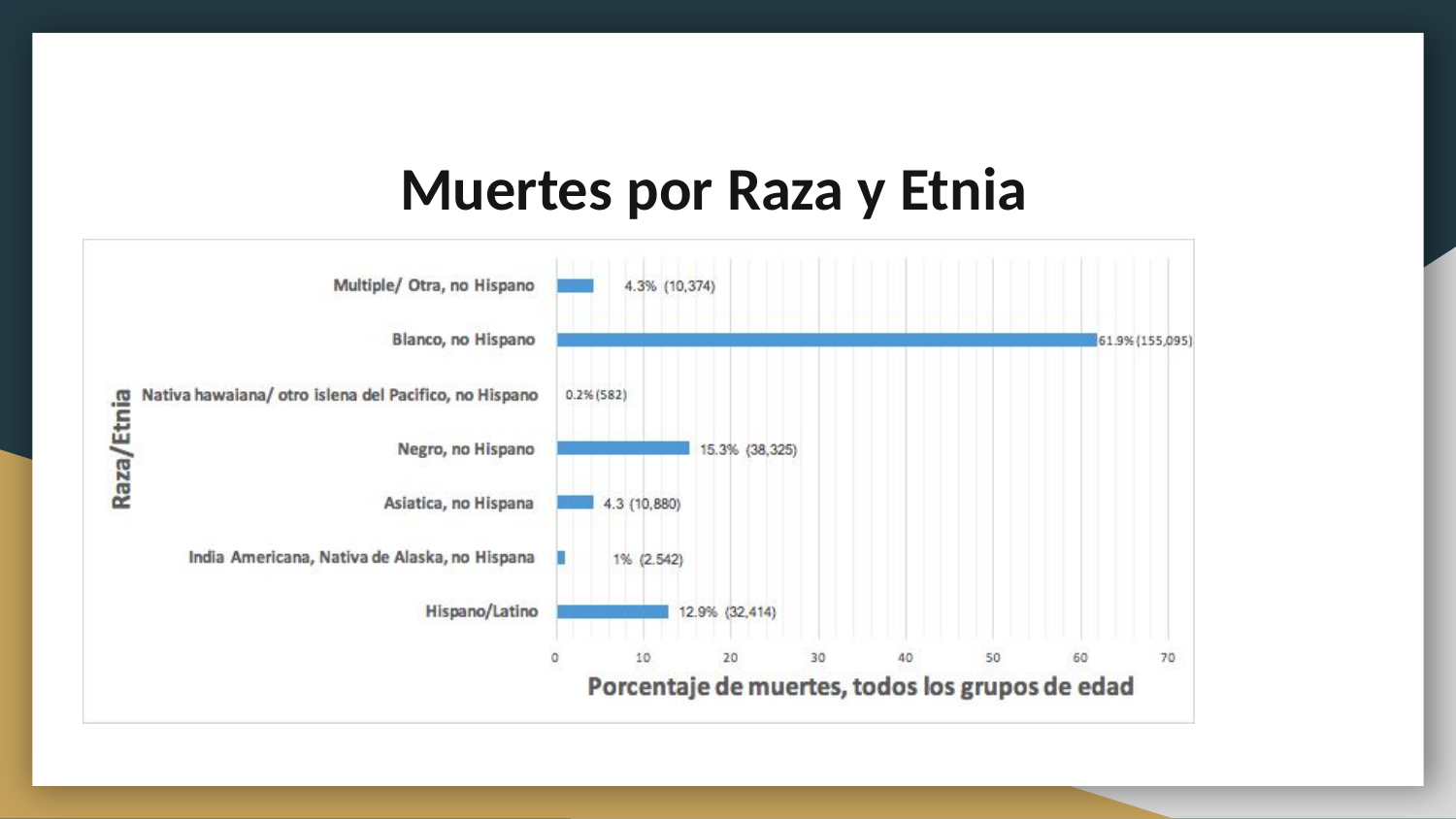

# Muertes por Raza y Etnia

## Slide 11
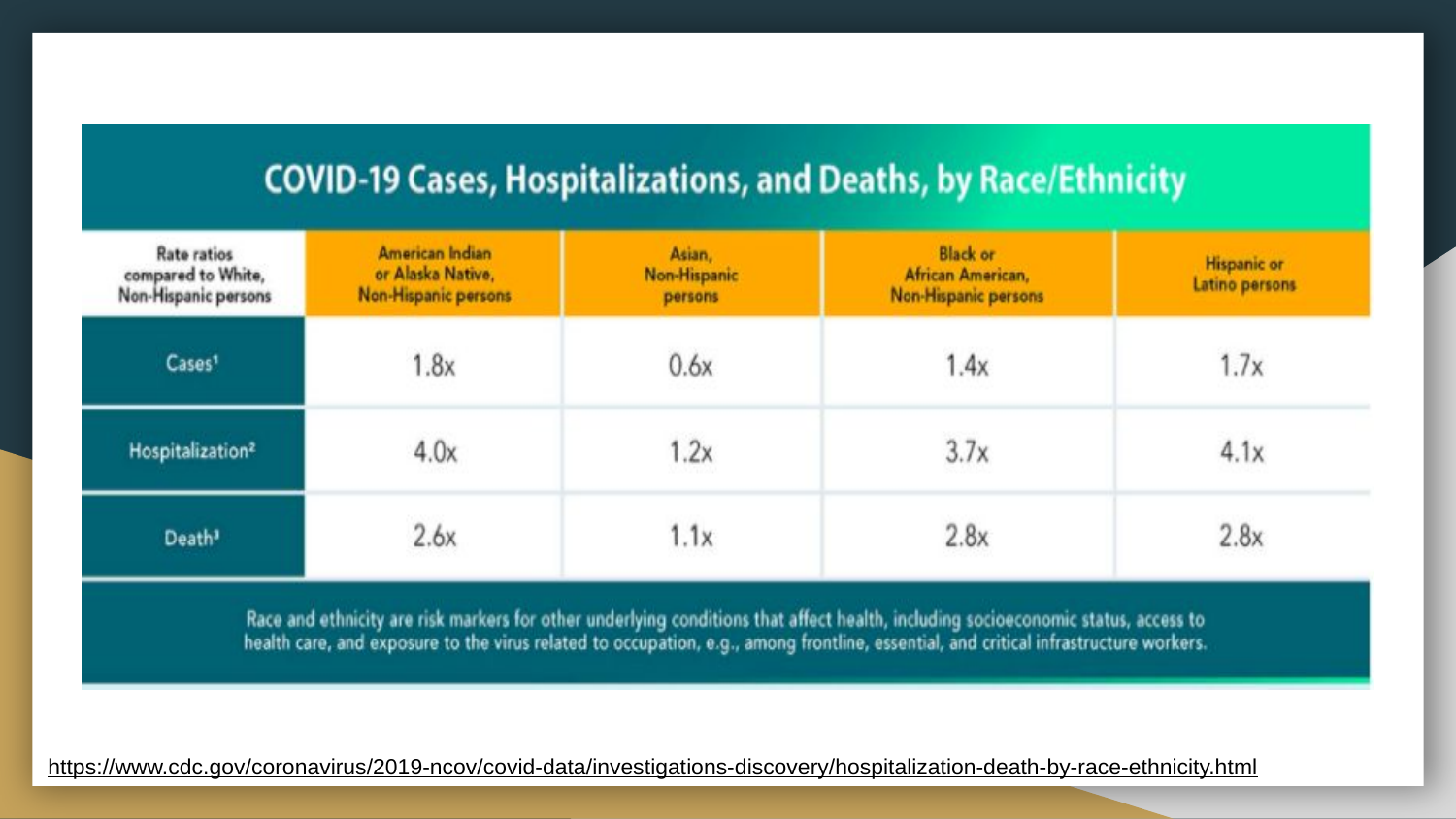

https://www.cdc.gov/coronavirus/2019-ncov/covid-data/investigations-discovery/hospitalization-death-by-race-ethnicity.html

## Slide 12
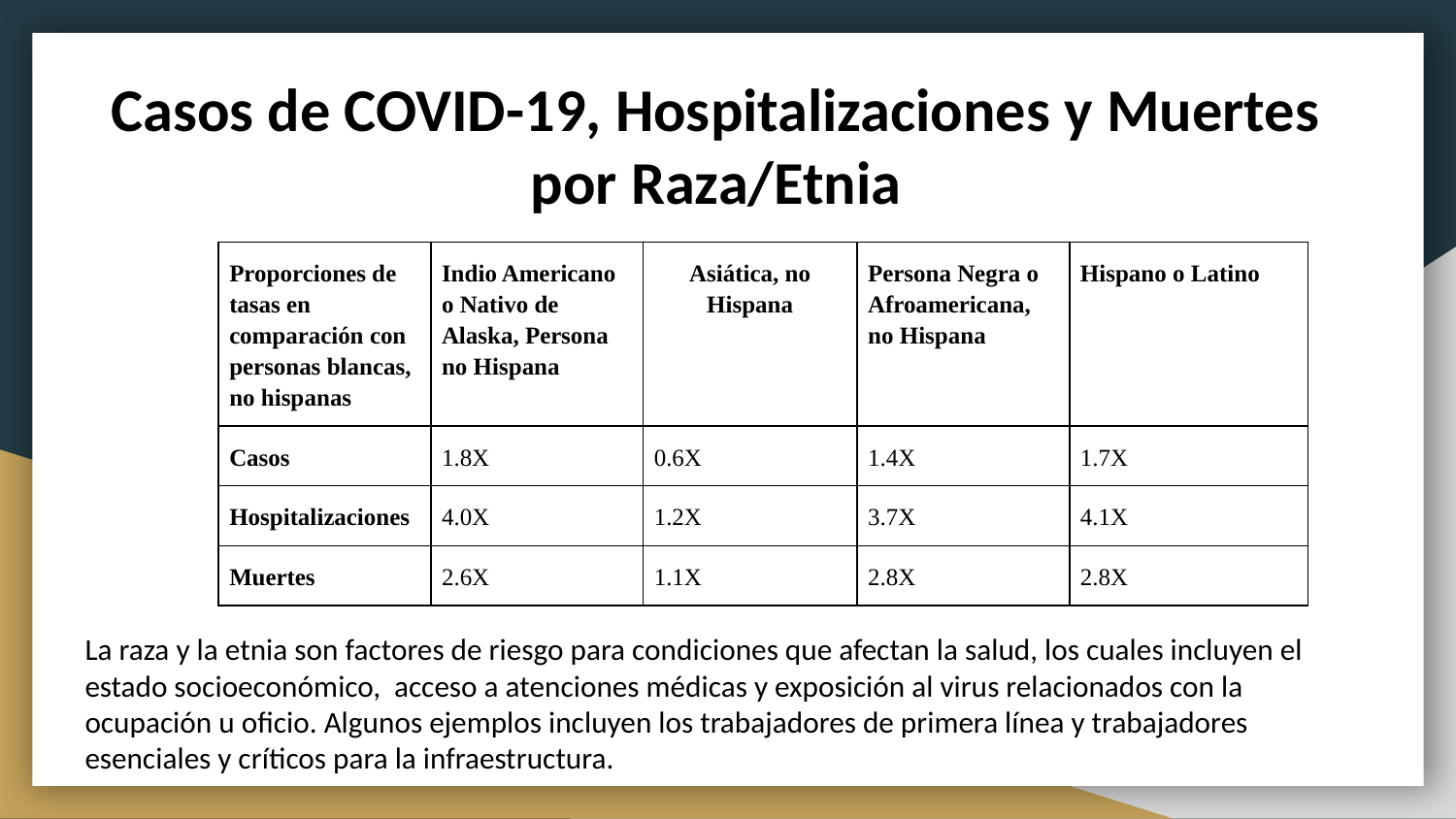

Casos de COVID-19, Hospitalizaciones y Muertes por Raza/Etnia
| Proporciones de tasas en comparación con personas blancas, no hispanas | Indio Americano o Nativo de Alaska, Persona no Hispana | Asiática, no Hispana | Persona Negra o Afroamericana, no Hispana | Hispano o Latino |
| --- | --- | --- | --- | --- |
| Casos | 1.8X | 0.6X | 1.4X | 1.7X |
| Hospitalizaciones | 4.0X | 1.2X | 3.7X | 4.1X |
| Muertes | 2.6X | 1.1X | 2.8X | 2.8X |
La raza y la etnia son factores de riesgo para condiciones que afectan la salud, los cuales incluyen el estado socioeconómico, acceso a atenciones médicas y exposición al virus relacionados con la ocupación u oficio. Algunos ejemplos incluyen los trabajadores de primera línea y trabajadores esenciales y críticos para la infraestructura.

## Slide 13
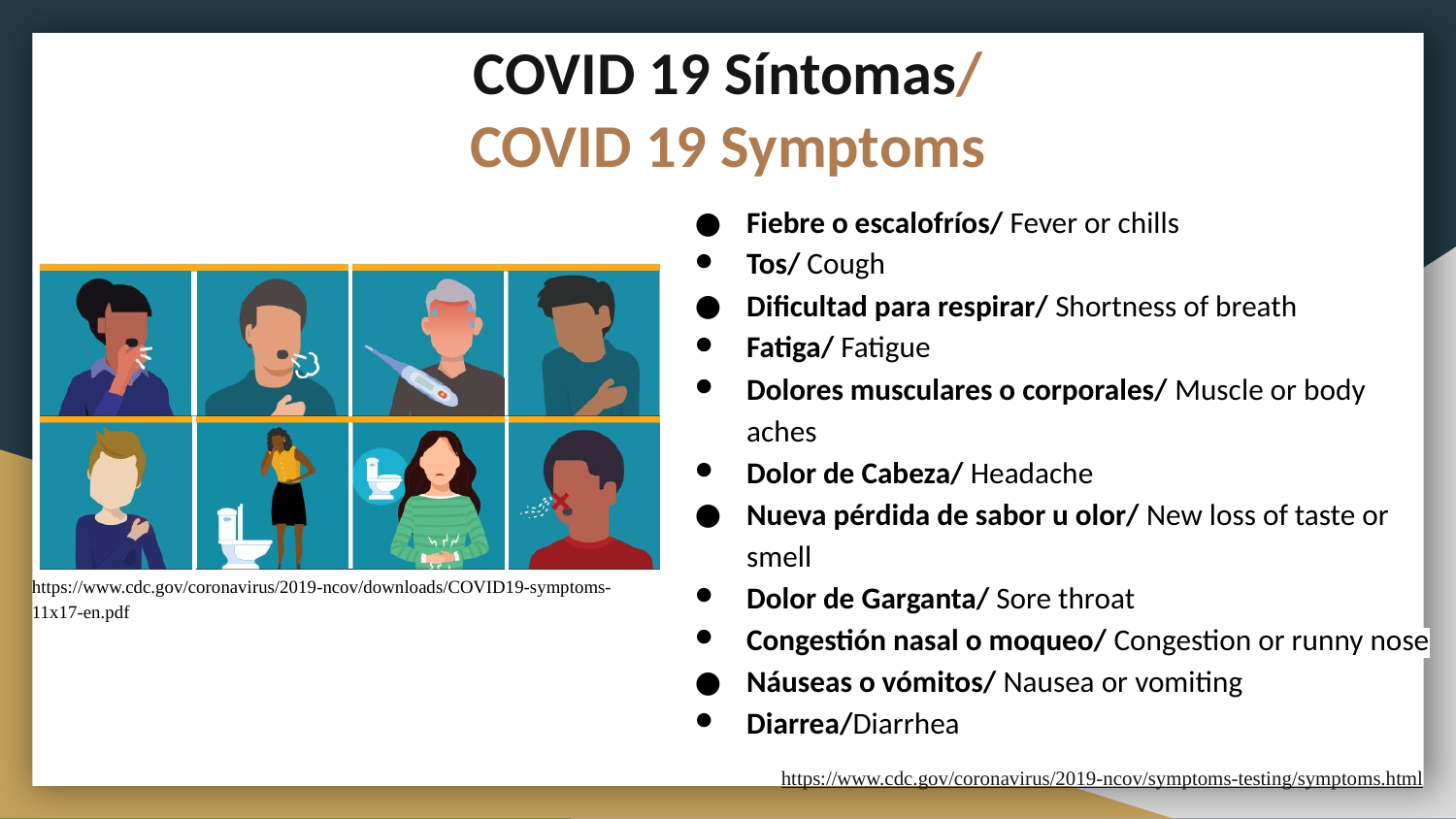

# COVID 19 Síntomas/
COVID 19 Symptoms
Fiebre o escalofríos/ Fever or chills
Tos/ Cough
Dificultad para respirar/ Shortness of breath
Fatiga/ Fatigue
Dolores musculares o corporales/ Muscle or body aches
Dolor de Cabeza/ Headache
Nueva pérdida de sabor u olor/ New loss of taste or smell
Dolor de Garganta/ Sore throat
Congestión nasal o moqueo/ Congestion or runny nose
Náuseas o vómitos/ Nausea or vomiting
Diarrea/Diarrhea
https://www.cdc.gov/coronavirus/2019-ncov/downloads/COVID19-symptoms-11x17-en.pdf
https://www.cdc.gov/coronavirus/2019-ncov/symptoms-testing/symptoms.html

## Slide 14
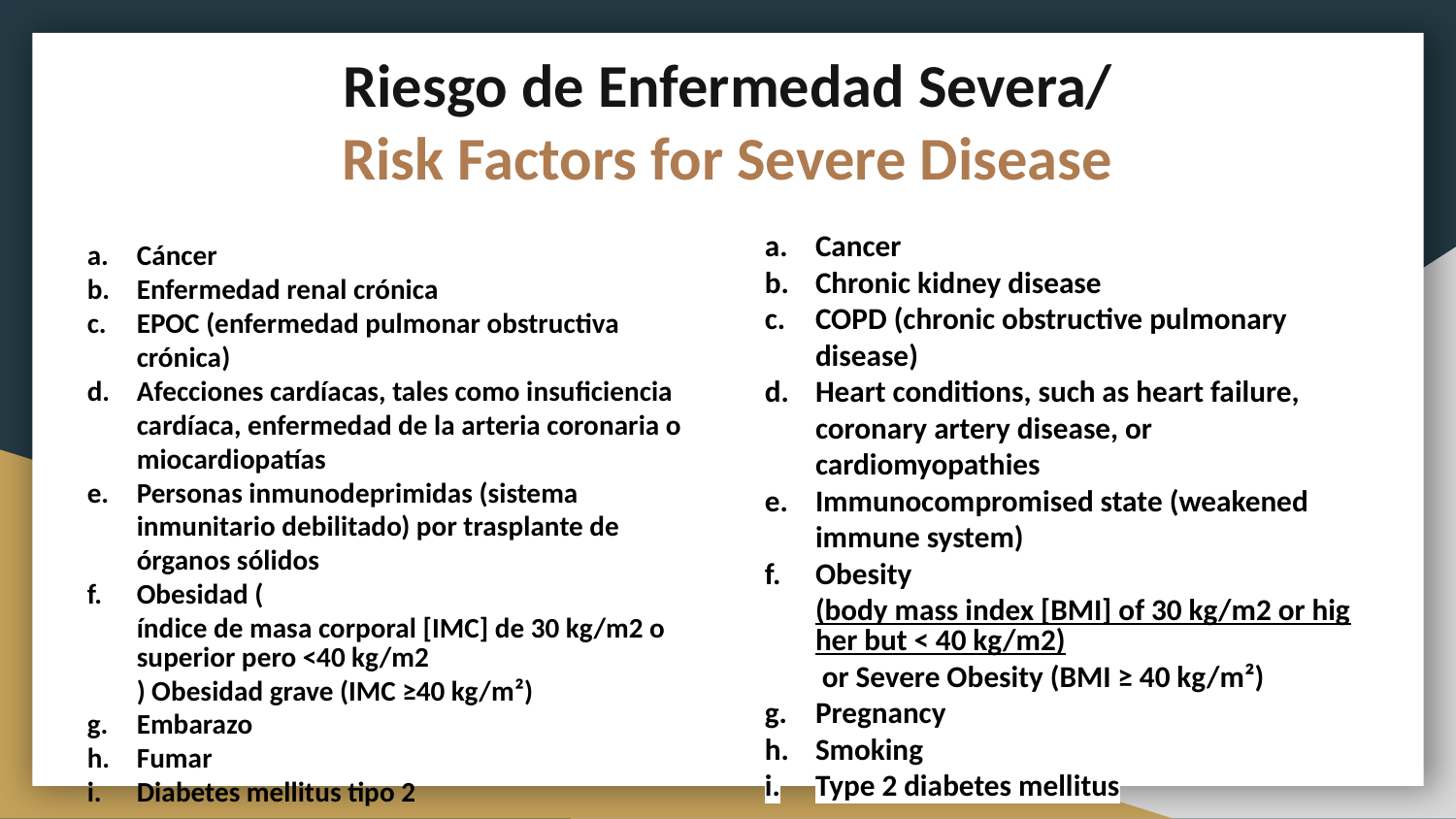

# Riesgo de Enfermedad Severa/ Risk Factors for Severe Disease
Cancer
Chronic kidney disease
COPD (chronic obstructive pulmonary disease)
Heart conditions, such as heart failure, coronary artery disease, or cardiomyopathies
Immunocompromised state (weakened immune system)
Obesity (body mass index [BMI] of 30 kg/m2 or higher but < 40 kg/m2) or Severe Obesity (BMI ≥ 40 kg/m²)
Pregnancy
Smoking
Type 2 diabetes mellitus
Cáncer
Enfermedad renal crónica
EPOC (enfermedad pulmonar obstructiva crónica)
Afecciones cardíacas, tales como insuficiencia cardíaca, enfermedad de la arteria coronaria o miocardiopatías
Personas inmunodeprimidas (sistema inmunitario debilitado) por trasplante de órganos sólidos
Obesidad (índice de masa corporal [IMC] de 30 kg/m2 o superior pero <40 kg/m2) Obesidad grave (IMC ≥40 kg/m²)
Embarazo
Fumar
Diabetes mellitus tipo 2

## Slide 15
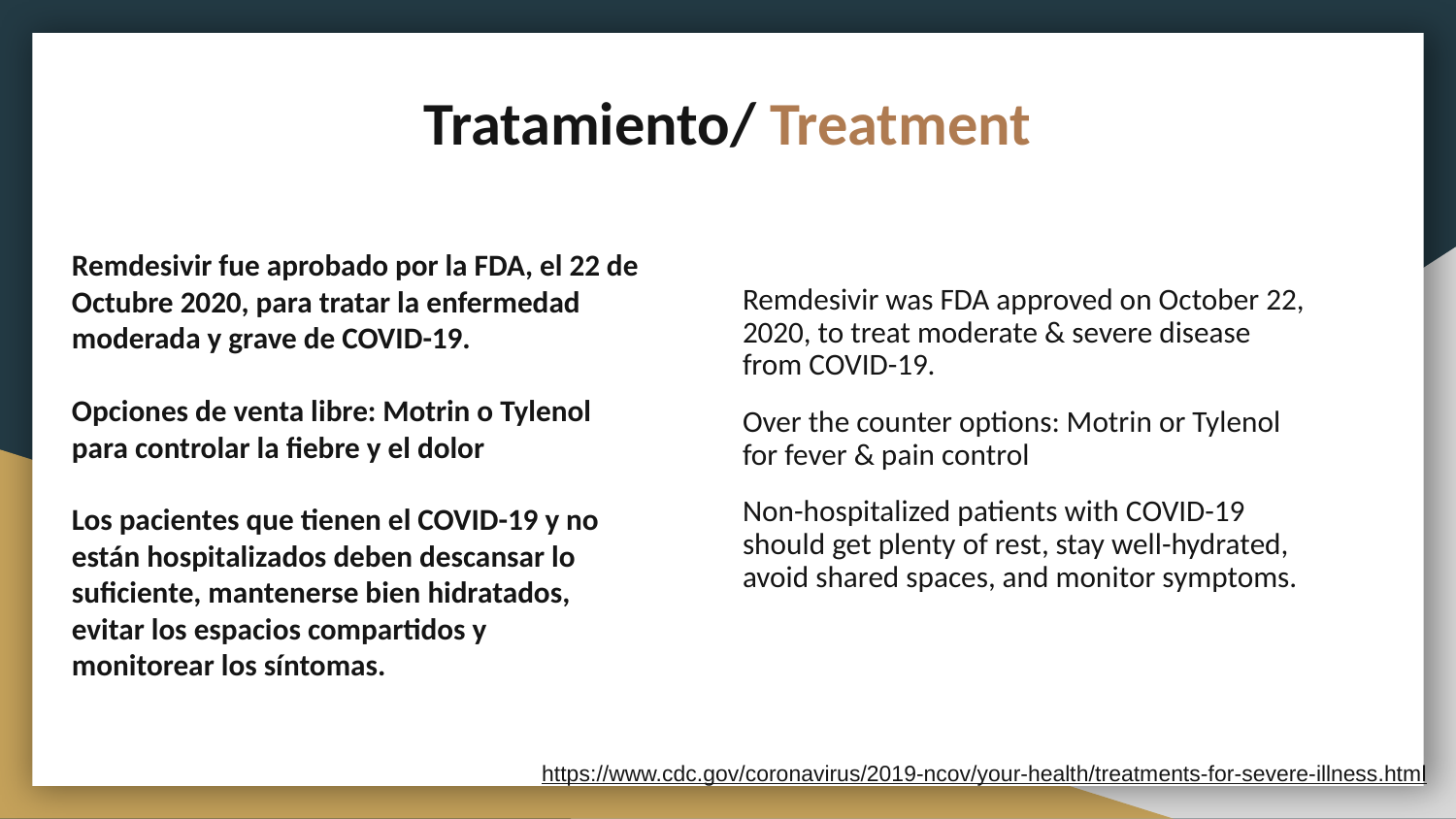

# Tratamiento/ Treatment
Remdesivir fue aprobado por la FDA, el 22 de Octubre 2020, para tratar la enfermedad moderada y grave de COVID-19.
Opciones de venta libre: Motrin o Tylenol para controlar la fiebre y el dolor
Los pacientes que tienen el COVID-19 y no están hospitalizados deben descansar lo suficiente, mantenerse bien hidratados, evitar los espacios compartidos y monitorear los síntomas.
Remdesivir was FDA approved on October 22, 2020, to treat moderate & severe disease from COVID-19.
Over the counter options: Motrin or Tylenol for fever & pain control
Non-hospitalized patients with COVID-19 should get plenty of rest, stay well-hydrated, avoid shared spaces, and monitor symptoms.
https://www.cdc.gov/coronavirus/2019-ncov/your-health/treatments-for-severe-illness.html

## Slide 16
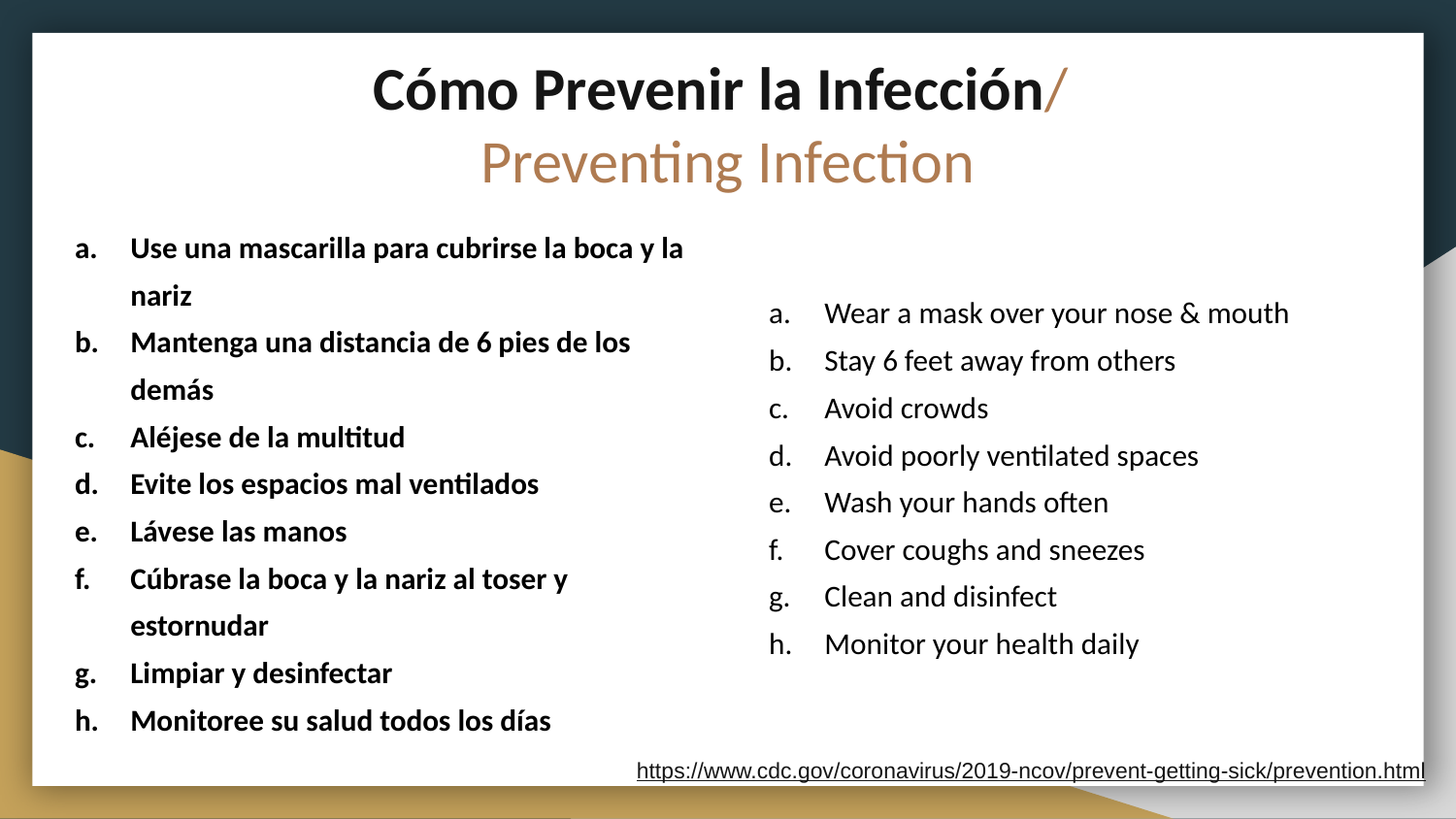

# Cómo Prevenir la Infección/  Preventing Infection
Use una mascarilla para cubrirse la boca y la nariz
Mantenga una distancia de 6 pies de los demás
Aléjese de la multitud
Evite los espacios mal ventilados
Lávese las manos
Cúbrase la boca y la nariz al toser y estornudar
Limpiar y desinfectar
Monitoree su salud todos los días
Wear a mask over your nose & mouth
Stay 6 feet away from others
Avoid crowds
Avoid poorly ventilated spaces
Wash your hands often
Cover coughs and sneezes
Clean and disinfect
Monitor your health daily
https://www.cdc.gov/coronavirus/2019-ncov/prevent-getting-sick/prevention.html

## Slide 17
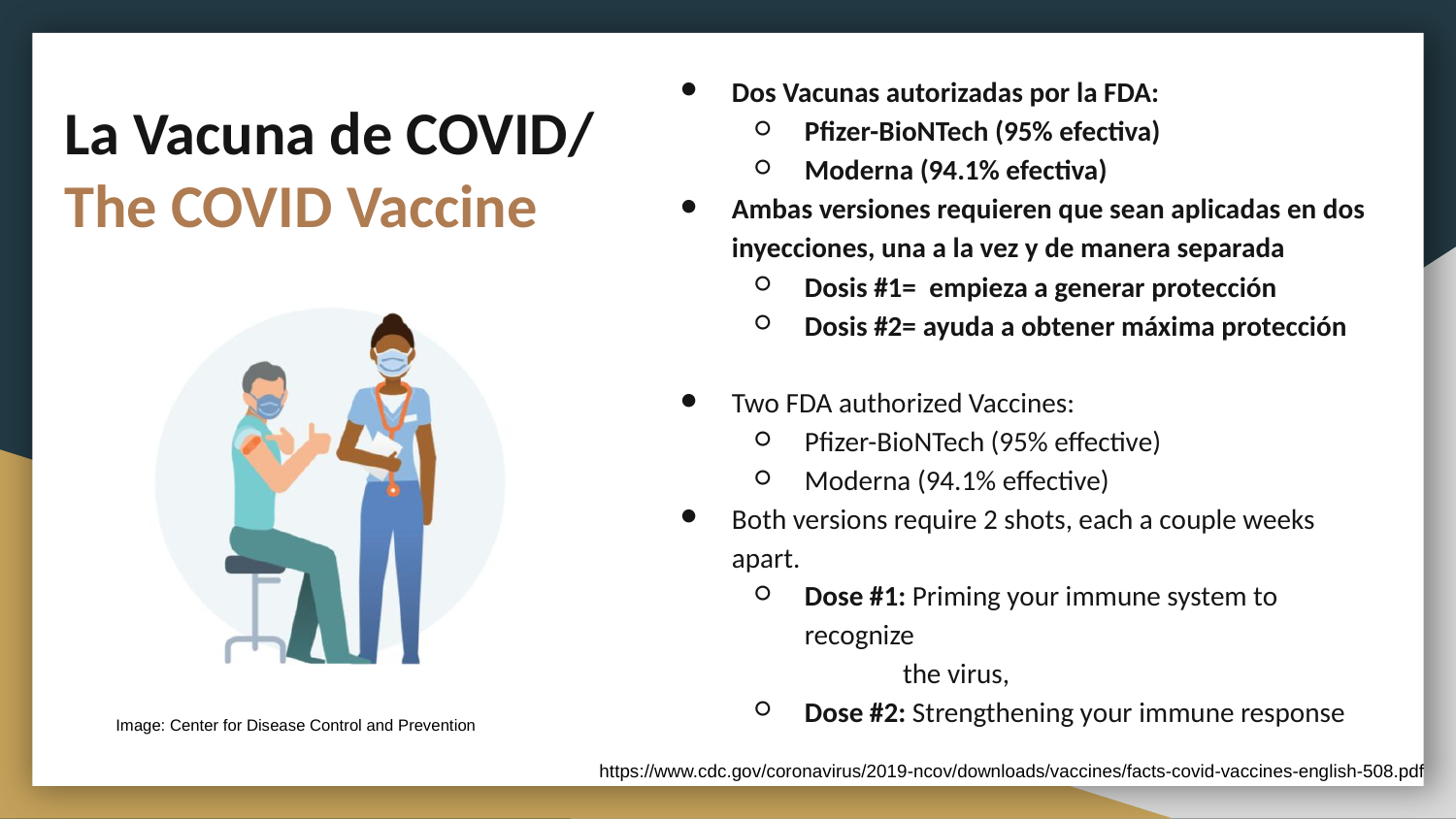

Dos Vacunas autorizadas por la FDA:
Pfizer-BioNTech (95% efectiva)
Moderna (94.1% efectiva)
Ambas versiones requieren que sean aplicadas en dos inyecciones, una a la vez y de manera separada
Dosis #1=  empieza a generar protección
Dosis #2= ayuda a obtener máxima protección
Two FDA authorized Vaccines:
Pfizer-BioNTech (95% effective)
Moderna (94.1% effective)
Both versions require 2 shots, each a couple weeks apart.
Dose #1: Priming your immune system to recognize
 the virus,
Dose #2: Strengthening your immune response
# La Vacuna de COVID/ The COVID Vaccine
Image: Center for Disease Control and Prevention
https://www.cdc.gov/coronavirus/2019-ncov/downloads/vaccines/facts-covid-vaccines-english-508.pdf

## Slide 18
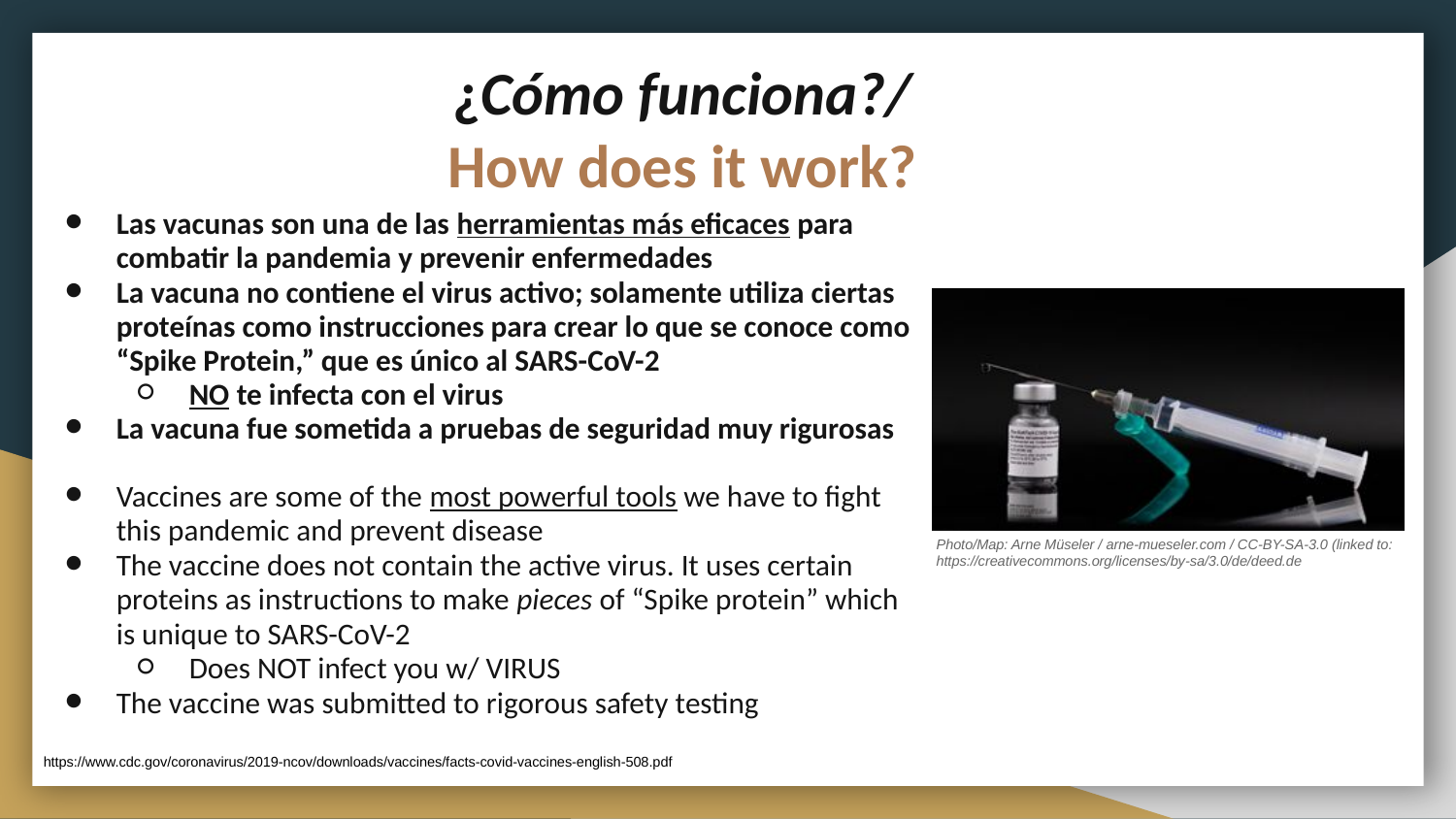

# ¿Cómo funciona?/  How does it work?
Las vacunas son una de las herramientas más eficaces para combatir la pandemia y prevenir enfermedades
La vacuna no contiene el virus activo; solamente utiliza ciertas proteínas como instrucciones para crear lo que se conoce como “Spike Protein,” que es único al SARS-CoV-2
NO te infecta con el virus
La vacuna fue sometida a pruebas de seguridad muy rigurosas
Vaccines are some of the most powerful tools we have to fight this pandemic and prevent disease
The vaccine does not contain the active virus. It uses certain proteins as instructions to make pieces of “Spike protein” which is unique to SARS-CoV-2
Does NOT infect you w/ VIRUS
The vaccine was submitted to rigorous safety testing
Photo/Map: Arne Müseler / arne-mueseler.com / CC-BY-SA-3.0 (linked to: https://creativecommons.org/licenses/by-sa/3.0/de/deed.de
https://www.cdc.gov/coronavirus/2019-ncov/downloads/vaccines/facts-covid-vaccines-english-508.pdf

## Slide 19
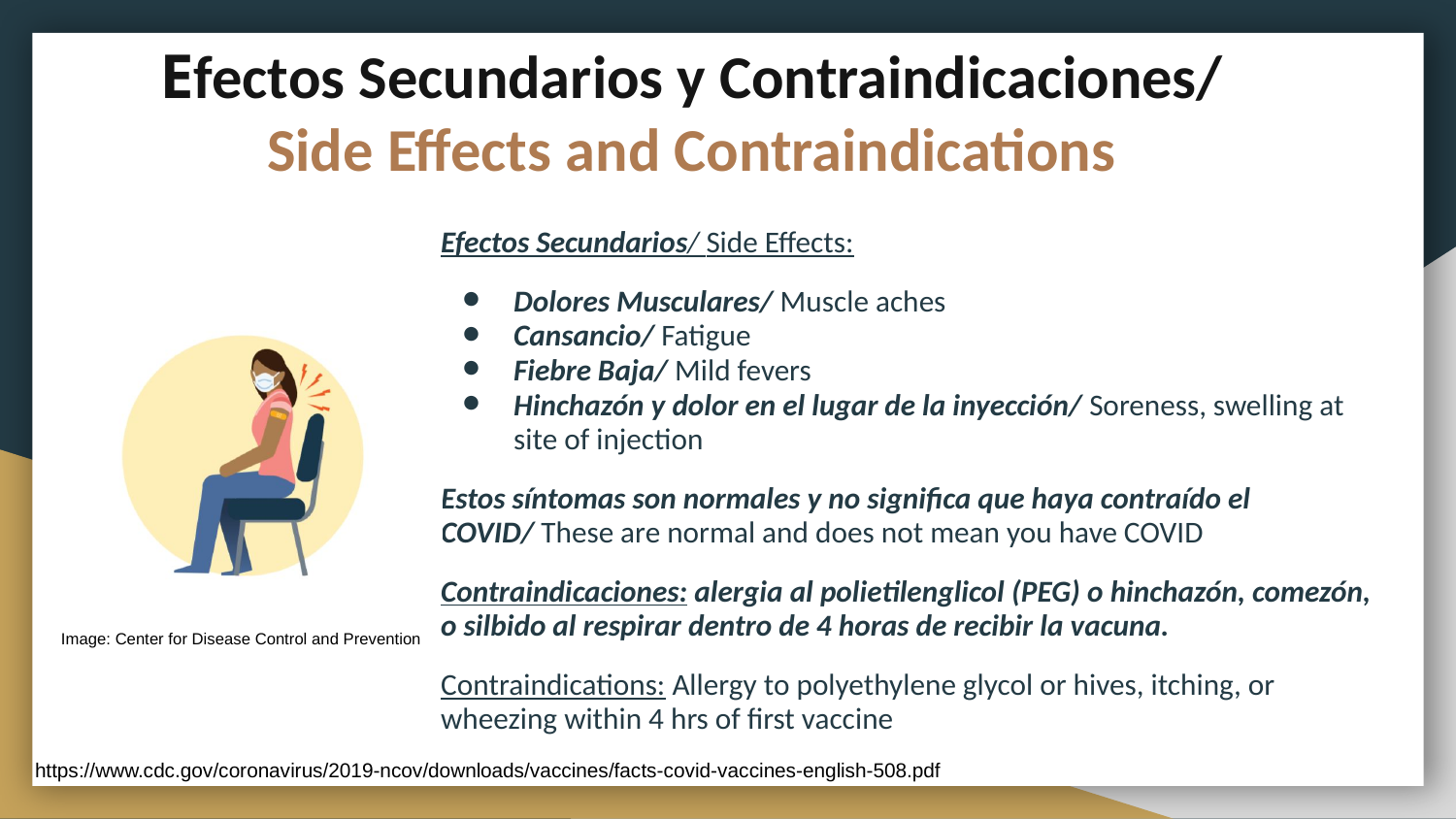

# Efectos Secundarios y Contraindicaciones/ Side Effects and Contraindications
Efectos Secundarios/ Side Effects:
Dolores Musculares/ Muscle aches
Cansancio/ Fatigue
Fiebre Baja/ Mild fevers
Hinchazón y dolor en el lugar de la inyección/ Soreness, swelling at site of injection
Estos síntomas son normales y no significa que haya contraído el COVID/ These are normal and does not mean you have COVID
Contraindicaciones: alergia al polietilenglicol (PEG) o hinchazón, comezón, o silbido al respirar dentro de 4 horas de recibir la vacuna.
Contraindications: Allergy to polyethylene glycol or hives, itching, or wheezing within 4 hrs of first vaccine
Image: Center for Disease Control and Prevention
https://www.cdc.gov/coronavirus/2019-ncov/downloads/vaccines/facts-covid-vaccines-english-508.pdf

## Slide 20
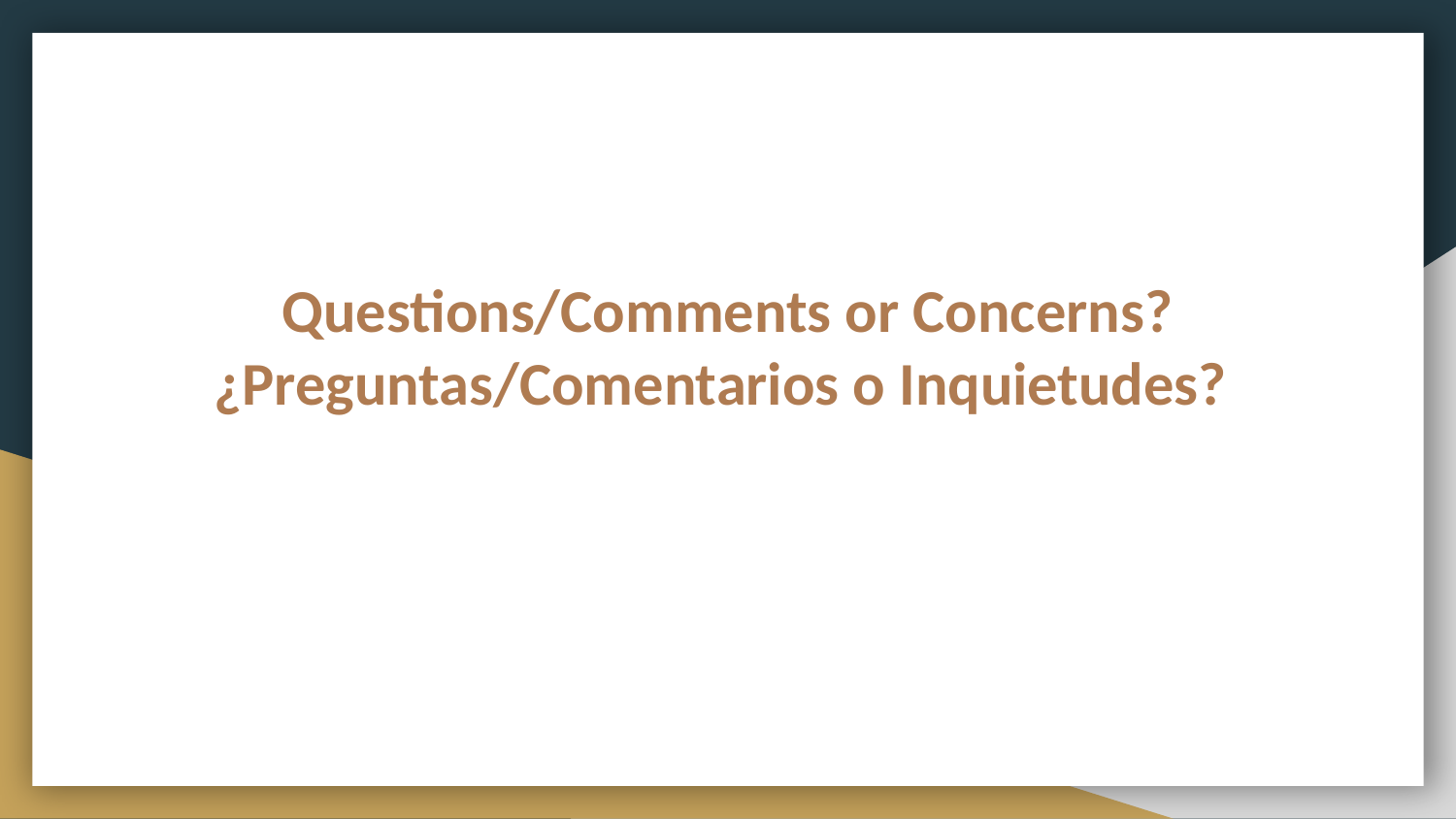

# Questions/Comments or Concerns?
¿Preguntas/Comentarios o Inquietudes?
